# Brain-stiffness-mimicking tilapia collagen gel promotes the induction of dorsal cortical neurons from human pluripotent stem cells

**DOI:** 10.1038/s41598-018-38395-5

**Published:** 2019-02-28

**Authors:** Misato Iwashita, Hatsumi Ohta, Takahiro Fujisawa, Minyoung Cho, Makoto Ikeya, Satoru Kidoaki, Yoichi Kosodo

**Affiliations:** 1 Korea Brain Research Institute, 61, Chemdan-ro, Dong-gu, Daegu, 41068 Republic of Korea; 2 Ihara & Co, Ltd, 3-263-23, Zenibako, Otaru, Hokkaido, 947-0261 Japan; 3 Institute for Materials Chemistry and Engineering, Kyushu University, 744 Motooka, Nishi-ku, Fukuoka, 819-0395 Japan; 4 Center for iPS Cell Research and Application (CiRA), Kyoto University, 53 Kawahara-cho, Shogoin, Sakyo-ku, Kyoto, 606-8507 Japan

## Abstract

The mechanical properties of the extracellular microenvironment, including its stiffness, play a crucial role in stem cell fate determination. Although previous studies have demonstrated that the developing brain exhibits spatiotemporal diversity in stiffness, it remains unclear how stiffness regulates stem cell fate towards specific neural lineages. Here, we established a culture substrate that reproduces the stiffness of brain tissue using tilapia collagen for *in vitro* reconstitution assays. By adding crosslinkers, we obtained gels that are similar in stiffness to living brain tissue (150–1500 Pa). We further examined the capability of the gels serving as a substrate for stem cell culture and the effect of stiffness on neural lineage differentiation using human iPS cells. Surprisingly, exposure to gels with a stiffness of approximately 1500 Pa during the early period of neural induction promoted the production of dorsal cortical neurons. These findings suggest that brain-stiffness-mimicking gel has the potential to determine the terminal neural subtype. Taken together, the crosslinked tilapia collagen gel is expected to be useful in various reconstitution assays that can be used to explore the role of stiffness in neurogenesis and neural functions. The enhanced production of dorsal cortical neurons may also provide considerable advantages for neural regenerative applications.

## Introduction

Determination of the fate of pluripotent stem cells and their development into functional cells is one of the crucial issues in the fields of developmental biology and regenerative medicine. Accumulating evidence demonstrates that biochemical factors, including exogenous gene transfer, regulate the fate determination of stem cells. Recent studies have also revealed the importance of the mechanical properties of the extracellular environment as a trigger of fate determination *in vitro*. Among these mechanical properties, the effect of extracellular stiffness on stem cell fate decisions has been intensively analyzed^1–6^. A number of studies have determined the range of tissue stiffness in muscle (10^4^ Pa), connective tissue (10^4^ Pa), skin (10^4^ to 10^5^ Pa) and bone (10^9^ to 10^10^ Pa)^7–9^. Notably, brain tissue showed lower stiffness, ranging from 10^2^ to 10^3^ Pa^7–14^. Despite these findings, little is known about how tissue stiffness affects the determination of stem cell fate towards specific cellular subtypes either *in vitro* or *in vivo*.

We previously reported the systematic analysis of living brain tissue stiffness during development^13^. Brain stiffness changes spatiotemporally throughout the embryonic stages during which neurogenesis from neural stem cells and construction of the brain architecture mostly occur. Accordingly, a fundamental question arises of whether neural lineage choice, including neural induction and differentiation, is influenced by differences in brain tissue stiffness. To address this question, it is essential to reproduce the stiffness conditions of living brain tissue *in vitro*. Several research groups have utilized polyacrylamide (PAA)-based hydrogels as a culture substrate to provide a wide range of stiffness^1–6,15–18^. However, it has proven technically demanding to consistently reproduce a substrate with a stiffness close to that of brain due to the relative softness^10–14^. Furthermore, acrylamide has been shown to be toxic to neurons^19^.

To overcome these difficulties, here we applied collagen as a material to produce a culture substrate mimicking the stiffness of brain tissue. Collagen is a major component of connective tissue in living tissue and can be easily extracted from various animals by acid solubilization. We selected collagen extracted from tilapia (*Oreochromis niloticus*) skin because the use of fish collagen rather than collagen of mammalian origin is expected to reduce the possibility of the transmission of animal diseases to humans (“zoonosis”)^20^. Although acid-soluble collagen molecules self-assemble into fibrils under physiological conditions, the stiffness of gels consisting of such fibrils can be controlled by chemical modification. 1-ethyl-3-(3-dimethylaminopropyl)-carbodiimide (EDC) is a commonly used crosslinking reagent for biomaterials, and N-hydroxysuccinimide (NHS) can enhance crosslinking between collagen fibers in the presence of EDC^21–25^. Here, we identified an effective combination of EDC and NHS that produces a consistently softer range of collagen gel that mimics brain tissue, the stiffness of which ranges from approximately 150 Pa close to the cortical plate^13^ to 1500 Pa close to the apical surface^14^. Notably, the chemically crosslinked collagen gel contained triple-helical structures that may provide a scaffold for integrin signaling. Furthermore, the gel showed high transparency, and its surface appeared almost flattened compared to fibril-formed collagen gels.

To test whether the gel established here can be used for stem cell culture and to evaluate the effect of stiffness on neural differentiation, we performed feeder cell-free cultures of human induced pluripotent stem cells (hiPSCs). HiPSCs cultured on gels showed impaired pluripotency and entered the neural lineage in a manner similar to cells cultured on plastic dishes. To our surprise, the neural characteristics acquired after 5 days of neural induction on a gel with a stiffness of 1500 Pa significantly switched to the lineage of dorsal forebrain neurons. These results demonstrate that the tilapia collagen gel established here can be utilized for neural induction from pluripotent cells and that exposure to gels of a particular stiffness during the initial stage of neural induction can determine the direction of differentiation towards a specific neural lineage.

## Results

### Characterization of tilapia skin collagen

**Figure 1:**
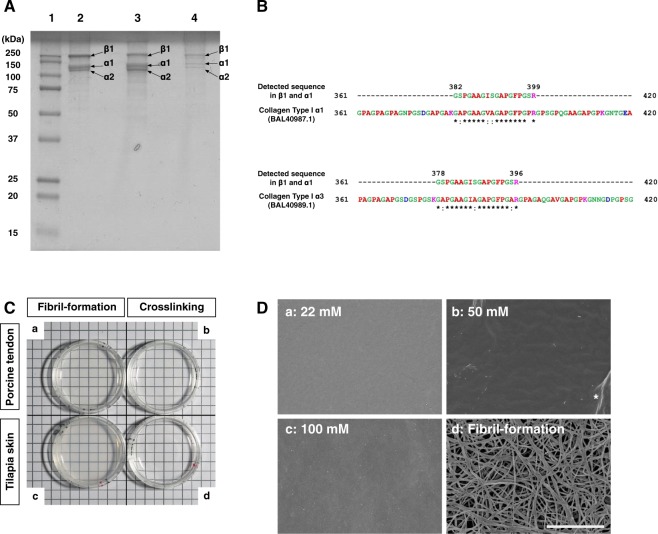
General characteristics of tilapia skin collagen. (**A**) SDS-PAGE analysis of collagen protein. Lane 1, molecular weight markers. Lane 2, tilapia skin collagen extracted in this study. Lanes 3 and 4, tilapia scale collagen and porcine tendon collagen, respectively. (**B**) Peptide sequence mapped on the reported sequence of tilapia collagen type I α1 and α3. Detected sequence in β1 (band No.1) and α1 (band No.2) shown in Fig. 1A and Supplementary Fig. S1B was identified as collagen type I α3, which is a fish specific collagen. BAL40987.1 and BAL40989.1 are tilapia collagen type I α1 and α3 in GenBank, respectively. Red, small and hydrophobic includes armomatic –Y; Blue, Acidic; Magenta, Basic –H; Green, Hydroxyl, sulfhydryl, amine and G; * (asterisk) indicates positions that have a single, fully conserved residue : (colon) indicates conservation between groups of strongly similar properties. (**C**) Transparency of gels prepared by various methods. The origin of the material was porcine tendon (a and b) or tilapia skin (c and d). The material shown in (a) and (c) was prepared by the fibril-formation method, and that shown in (b) and (d) was prepared by the crosslinking method. Gels prepared by the crosslinking method displayed higher transparency than those prepared by the fibril-formation method. (**D**) SEM images of the surfaces of tilapia collagen gels prepared by the crosslinking ([NHS]/[EDC] = 0.1, a to c) and fibril-formation (d) methods are shown. The final concentration of EDC is indicated in each panel. Note that gels prepared by the crosslinking method displayed nearly flat surfaces, whereas distinct fibrous structures were observed in gels prepared using the fibril-formation method. The asterisk indicates creases that appeared during the preparation of the sample for SEM observation. Bar = 3 μm.

Tilapia skin collagen was extracted by acid solubilization, and SDS-PAGE was used to characterize the polypeptide composition of the tilapia skin collagen (Fig. 1A and Supplementary Fig. S1A). Tilapia skin collagen exhibited two distinct bands between 100 and 150 kDa and a prominent band near 250 kDa without any major impurities (Fig. 1A, lane 2). According to previous reports, the molecular weight of the α1 and 2 chains of collagen is approximately 116.5 to 126.2 kDa, and that of the β chain is 255.5 to 281.1 kDa^26–28^. The molecular weights of the polypeptides present in extracted tilapia skin collagen fell within this range. The band pattern resembled that produced by commercially available type I collagen extracted from tilapia scales and porcine tendon (Fig. 1A, lanes 3 and 4, respectively). To characterize collagen used in this study, we performed mass spectrometry using MALDI-TOF/MS analysis (Fig. 1B and Supplementary Fig. S1B). We obtained the several peptide fragments and found matched protein sequences in the protein database (Supplementary Table S1). In addition, fish specific collagen type I α3^29^ was identified as Sequence_2 in the β and α1 bands (Supplementary Fig. S1Bb and Table S1). For further analysis of this sequence, we compared this sequence with fish collagen type I α1, 2 and 3. We found the matched the position of this sequence in tilapia collagen type I α1 and 3 (Fig. 1B). This result was consistent with the previous report that collagen type I α3 forms a β chain together with α1 and 2 and falls with α1 in SDS-PAGE^29^. Taken together with these results, collagen extracted from tilapia skin can be categorized as a type I collagen.

To test whether tilapia skin collagen can form gels, we first attempted various gel preparation methods in which we compared collagen from tilapia skin with collagen obtained from porcine tendon (Fig. 1C and Supplementary Fig. S1C). Collagen gel prepared by neutralization (hereafter, the “(physical) fibril-formation method”) of either porcine tendon or tilapia skin collagen displayed a muddied white color in both cases (Fig. 1Ca,c, respectively). In contrast, a gel prepared from either porcine tendon or tilapia skin collagen by adding crosslinking reagents (hereafter, the “(chemical) crosslinking method”) had a clear appearance in both cases (Fig. 1Cb and d, respectively). These results demonstrate that collagen from tilapia skin can form a gel in a manner that is similar to gel formation by commercially available porcine tendon collagen. To further analysis of gel characteristics, surface of gels was examined using scanning electron microscopy (SEM) because the surface structure of the culture substrate can influence the determination of cell fate^30–32^. Remarkably, the surfaces of gels prepared by the chemical crosslinking method were almost flat, and no detectable fiber-like structures were present irrespective of the concentrations of crosslinking solution used in the preparation of the gels (Fig. 1Da–c). In contrast, distinct fiber-like structures were observed in the gels prepared by the fibril-formation method (Fig. 1Dd). These results show that the surfaces of tilapia skin collagen gels established by the crosslinking method do not contain structures that may affect cell fate determination.

### Effect of chemical crosslinkers on the mechanical properties of tilapia collagen gels

To achieve variety in the stiffness of the gels, we next investigated the concentration of crosslinking reagents. For this purpose, we mixed EDC and NHS, two widely used soluble crosslinking reagents, with tilapia skin collagen at various concentrations. Crosslinking by EDC activates the carboxylic acid group in collagen. NHS forms a stable ester and increases the number of crosslinking sites between collagen fibrils in the presence of EDC^33,34^. Importantly, a byproduct of this reaction is urea, which is not hazardous to cells and is easily removed by washing. Therefore, we chose EDC and NHS as crosslinking reagents.

**Figure 2:**
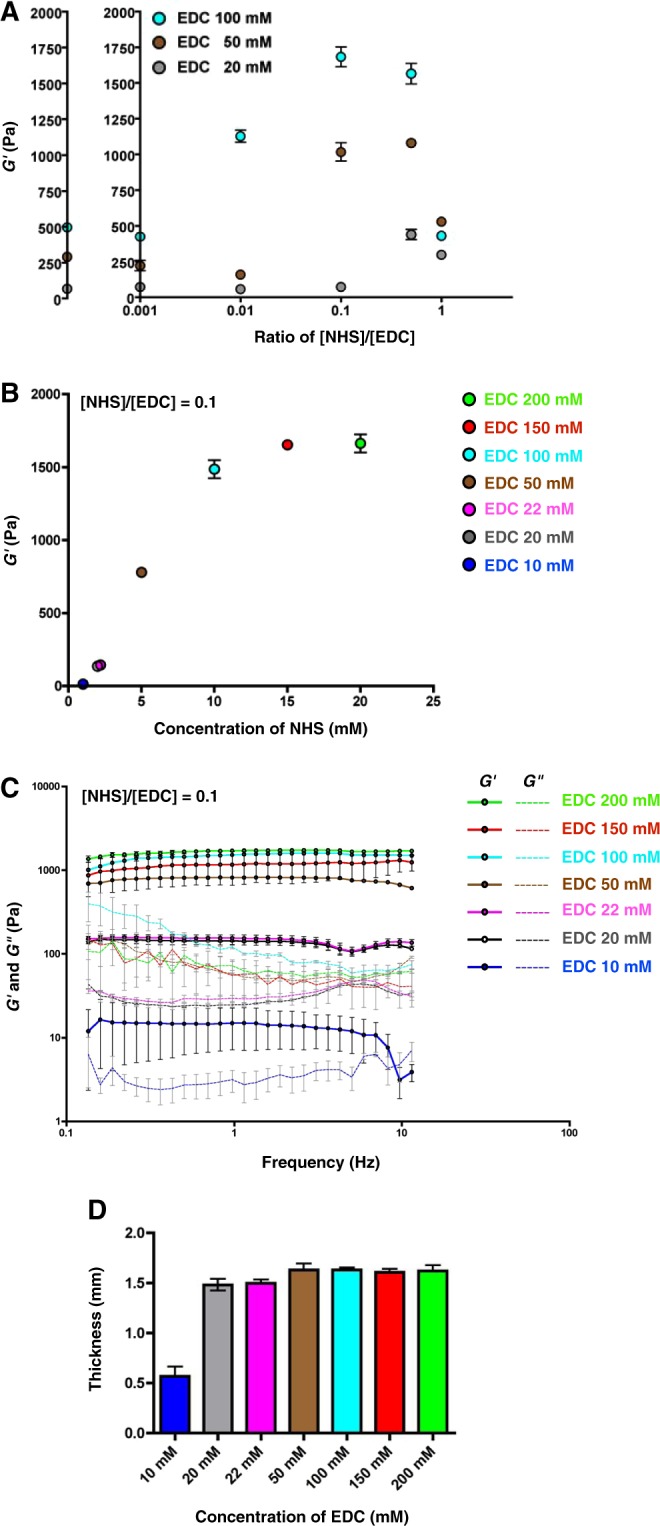
Effects of EDC and NHS concentrations on gel stiffness. (**A**) Storage modulus (*G*′) of gels prepared using various combinations of EDC and NHS. The storage modulus of gels without NHS was approximately 500 Pa at 100 mM EDC. When NHS solution was added, the storage modulus showed a linear increase over the range 0.001 to 0.1 ([NHS]/[EDC]). The linearity was no longer present at ratios of 0.5 and 1. The widest range of storage modulus was observed at a ratio of 0.1. (**B**) Storage modulus of gels as a function of the concentration of crosslinkers at a ratio of [NHS]/[EDC] of 0.1. The storage modulus showed linearity between 20 and 100 mM EDC; the value of the storage modulus saturated at approximately 1600 Pa at the highest concentrations of EDC (150 and 200 mM). (**C**) Frequency-dependent storage and loss modulus of gels. All conditions with the exception of 10 mM of EDC showed stable storage modulus values during measurement. Solid lines and dashed lines indicate the storage modulus (*G*′) and the loss modulus (*G*″), respectively. (**D**) Thickness of the gels. All conditions except 10 mM EDC resulted in the formation of a gel of approximately 1.5 mm thickness when 2 ml of solution was added to a 35-mm dish.

The gel stiffness was defined by the storage modulus of the gel (*G*′) as shown in Fig. 2A. In the absence of NHS, the average value of *G*′ increased up to 496 ± 6 Pa at 100 mM EDC depending on the EDC concentration (67 ± 3, 290 ± 13 and 496 ± 6 Pa at 20, 50 and 100 mM EDC, respectively).

Because it provided the broadest range of stiffness, we used an [NHS]/[EDC] ratio of 0.1 in further attempts. We tested gel stiffness with seven combinations of [NHS] and [EDC] at a ratio of 0.1 (Supplementary Table S3). The average values of *G*′ observed were 13.0 ± 1 [EDC 10 mM]_0.1_, 136 ± 13 [EDC 20 mM]_0.1_, 145 ± 14 [EDC 22 mM]_0.1_, 780 ± 17 [EDC 50 mM]_0.1_, 1487 ± 62 [EDC 100 mM]_0.1_,1654 ± 27 [EDC 150 mM]_0.1_ and 1663 ± 62 Pa [EDC 200 mM]_0.1_ (Fig. 2B and Supplementary Table S3). The *G*′ value increased linearly with the EDC concentration over the range 20 to 100 mM and reached a plateau at concentrations greater than 150 mM (Fig. 2B).

Analysis of the frequency-dependent storage moduli of the gels demonstrated the stability of gels within the range [EDC 20 mM]_0.1_ to [EDC 200 mM]_0.1_. To further investigate the properties of gels, we measured the *G*′ and loss modulus (*G*″) with a linear strain condition. We found that the *G*″, indicating viscosity of gels, was maintained lower than *G*′ during measurement in all the conditions with the exception of EDC 10 mM (Fig. 2C and Supplementary Fig. S2). In addition, we measured the time dependency of *G*′ and *G*″ to confirm the polymerization kinetics during crosslinking (Supplementary Fig. S3). The *G*′ value in 22 and 100 mM was lower than *G*″ at the beginning of the measurement. However, the *G*′ value crossed over the *G*″ value immediately and remained higher value than *G*″ during measurement. These results suggested that crosslinking was dominant during gelation and contributes to maintain gel elasticity within the range of 22 to 100 mM of EDC. In contrast, gels prepared at the lowest concentration [EDC 10 mM]_0.1_ showed great dispersion during measurement and exhibited lower *G*′ value than *G*″ during the later phase of measurement (Fig. 2C and Supplementary Fig. S2). We also found that the thickness of gels prepared at [EDC 10 mM]_0.1_ was significantly decreased (Fig. 2D), indicating that the gel’s internal structure was unstable at lower concentrations of EDC and NHS.

### Molecular properties of collagen during gelation

**Figure 3:**
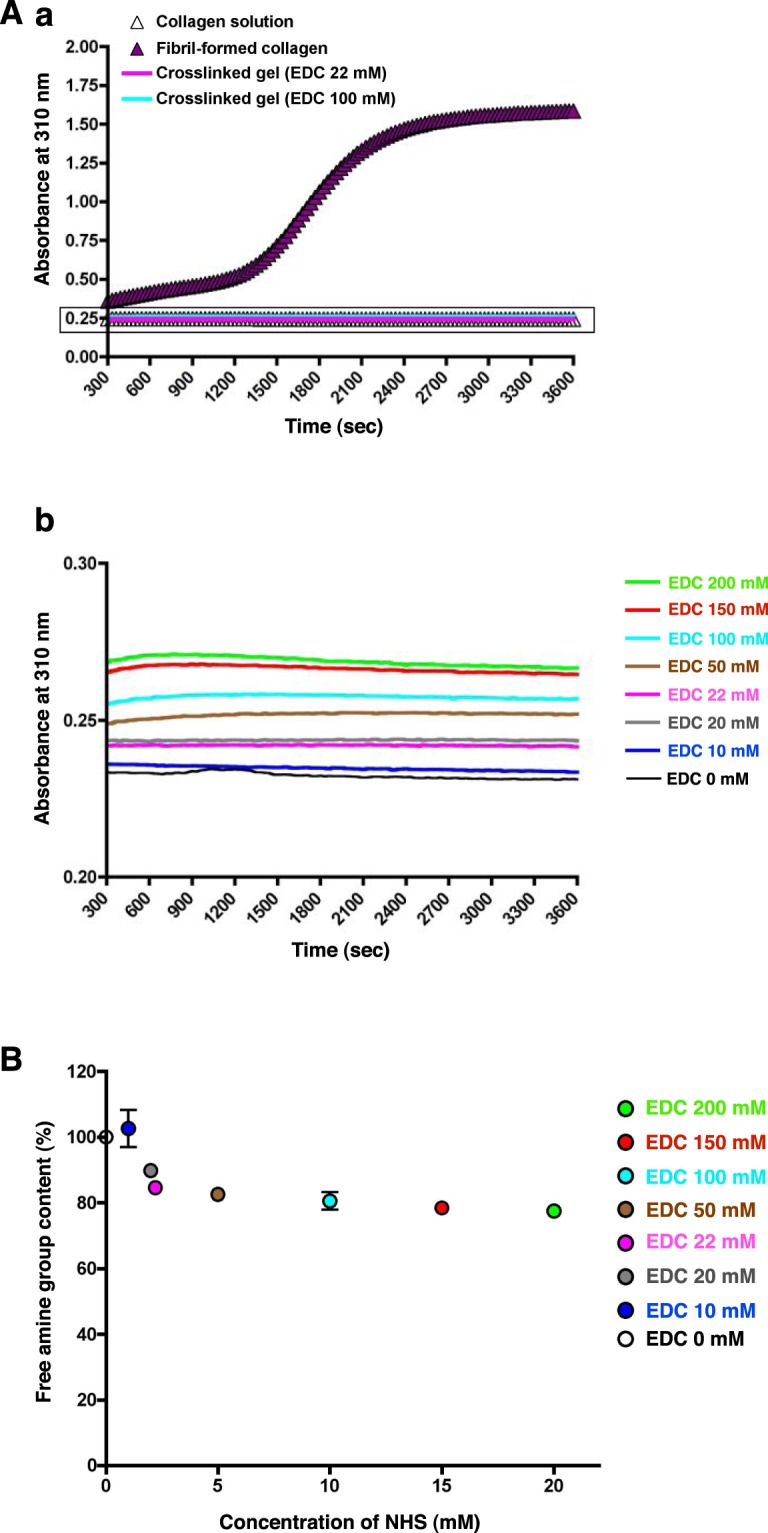
Molecular properties of collagen during gelation. (**A**) (a) A typical time plot of turbidity during gelation of a collagen solution (white triangles), fibril-formation method (purple triangles) and crosslinkers at an [NHS]/[EDC] ratio of 0.1 (22 and 100 mM EDC (pink and blue lines). The absorbance at 310 nm gradually increased and reached a plateau, displaying a sigmoid curve pattern in the fibril-formation method, whereas it was maintained at a constant value in the collagen solution and in gels prepared by the crosslinking method. (b) Magnified view of the rectangle area in (a) showing absorbance at 310 nm when various EDC and NHS concentrations were used in the crosslinking method. (**B**) The degree of crosslinking obtained at various EDC and NHS concentrations was monitored by measurement of the free amine group content of the gels. The data were plotted over a time period of 300 sec.

In further analysis of the characteristics of collagen in gels, we focused on the behavior of collagen molecules during gelation. First, we investigated the interaction between collagen fibers during gelation by monitoring the turbidity of the collagen solution at 310 nm (Fig. 3A). In fibril-formed collagen, the absorbance at 310 nm increased and reached a plateau (Fig. 3Aa, purple triangle). Figure 3Ab shows a magnified view of the representative plot shown in the rectangle in Fig. 3Aa. Compared to gels prepared using the fibril-formation method, absorbance at 310 nm was significantly suppressed in gels prepared by the chemical crosslinking method (Fig. 3Aa,Ab); these gels displayed absorbance that was similar to that of the control collagen solution (white triangle in Fig. 3Aa). Second, we examined the degree of crosslinking by measuring the free amine group content of the gels (Fig. 3B). Compared to the control without EDC and NHS, the free amine group content decreased from 100% to approximately 80% over the range [EDC 22 mM]_0.1_ to [EDC 200 mM]_0.1_, whereas the free amine group content was higher at [EDC 10 mM]_0.1_ and [EDC 20 mM]_0.1_. Taken together, these results show that the gels prepared by the crosslinking method showed elastic properties with a high linearity along EDC concentration within the range [EDC 20 mM]_0.1_ to [EDC 100 mM]_0.1_ and sufficient crosslinking reaction within the range [EDC 22 mM]_0.1_ to [EDC 200 mM]_0.1_. Therefore, we pursued further characterization of gels prepared at [EDC 22 mM]_0.1_ to [EDC 100 mM]_0.1_ because these EDC concentrations resulted in *G*′ values ranging from 150 to 1500 Pa (close to the stiffness of brain tissue) in an accurately adjustable manner.

**Figure 4:**
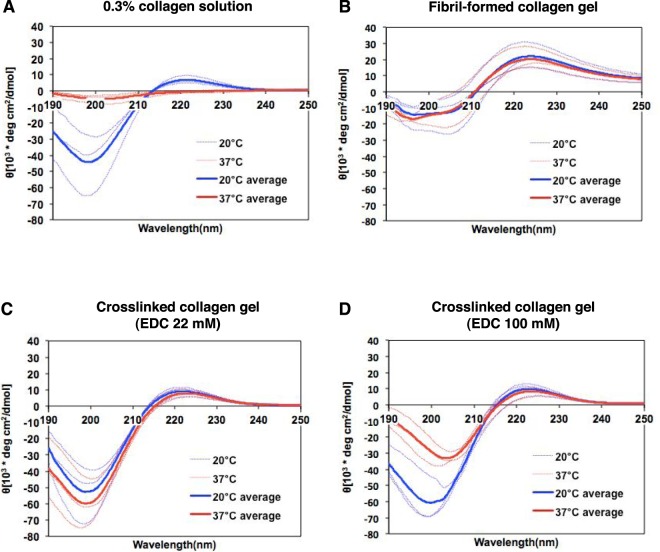
CD spectra of tilapia collagen solution and gels. The CD spectra of the following materials were measured at 20 °C (gel-forming temperature, shown in blue) and at 37 °C (culture temperature, shown in red) (n = 3 for each condition): (**A**) 0.3% tilapia collagen solution diluted in HCl (pH 3.0); (**B**) fibril-formed physical collagen gel; (**C,D**) chemically crosslinked collagen gels (SOFT [EDC 22 mM]_0.1_ and HARD [EDC 100 mM]_0.1,_ respectively). The thin dotted lines indicate the individual spectra; the bold lines indicate the average spectral values for each condition.

We measured the circular dichroism (CD) spectrum of the gel material to determine whether a collagen triple helix was present in the gels. We used four representative conditions for CD spectral analysis at 20 °C and 37 °C: 0.3 wt% tilapia collagen solution (pH 3.0), fibril-formed physical collagen gels, and chemically crosslinked collagen gels prepared with 22 and 100 mM EDC ([EDC 22 mM]_0.1_ and [EDC 100 mM]_0.1_, respectively) (Fig. 4). A positive peak at 221 nm and negative peaks near 190–200 nm reflecting the presence of a triple collagen helix were observed at 20 °C under all conditions; these peaks disappeared in 0.3 wt% collagen solution at 37 °C (Fig. 4A). The pattern of positive peaks at 221 nm in the [EDC 22 mM]_0.1_ and [EDC 100 mM]_0.1_ samples was essentially similar under the tested conditions of crosslinker concentration and temperature (Fig. 4C,D). The wavelengths and heights of the negative peaks varied depending on the gel conditions. These results show that tilapia collagen gels prepared by the chemical crosslinking method contain triple-helical collagen that is stably maintained under the physiological conditions used to culture mammalian cells.

### Culture of human iPSCs on tilapia collagen gels

**Figure 5:**
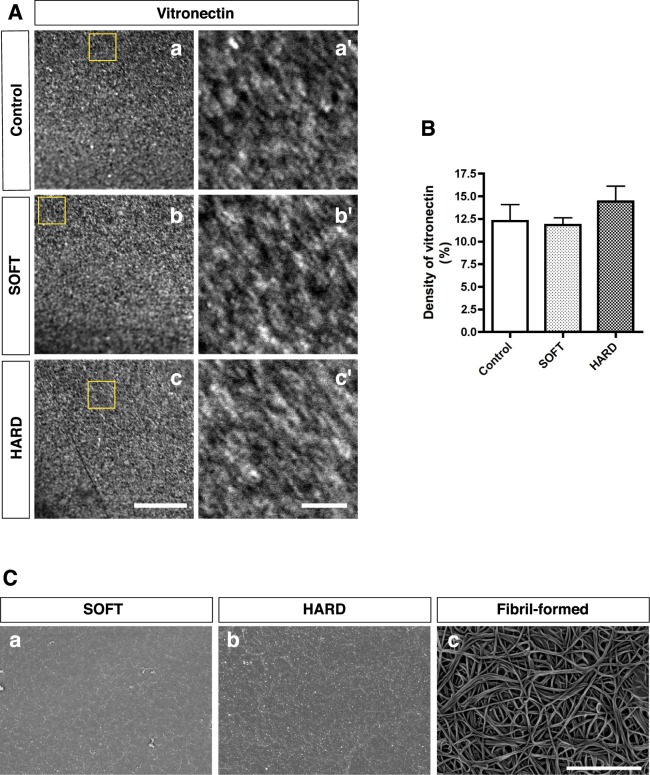
Quantification of ligand distribution on culture substrate. (**A**) Vitronectin distribution on culture substrate using anti-vitronectin antibody (White particles in a, b and c). Magnified images indicated as yellow squares (a′, b′ and c′). Bars = 10 μm for a, b and c and 2 μm for a′, b′ and c′. (**B**) Quantification of density of vitronectin. There was no significant difference in the density of vitronectin (one-way ANOVA and Turkey *post hoc* test). (**C**) SEM images of the surface structures of gels coated with Vitronectin XF (SOFT, HARD and fibril-formed collagen gel) are shown. Similar particles were observed after coating irrespective of the stiffness of chemically crosslinked gels (a and b). Note that the fibrous structure of the fibril-formed collagen gel was retained after coating (c). Bar = 3 μm.

Considerable evidence has been presented indicating that several types of undifferentiated stem cells tend to differentiate into neural lineages when they are cultured on soft substrates^2–6,16^. Here, we addressed whether tilapia collagen gels that mimic brain stiffness have an effect on the differentiation of pluripotent stem cells. HiPSCs were maintained in plastic dishes without feeder cells to avoid the stiffness effect of feeder-derived matrices^35^. Subsequently, hiPSC-colonies were enzymatically dissociated into single-cell suspensions and seeded at equal densities for passaging either on plastic dishes or on chemically crosslinked gels; the medium was then changed to a neural differentiation medium that has been reported to result in a high rate of production of dorsal cortical neurons^36^. For hiPS cell-line, we chose RPChiPS771-2 because of the higher production rate of dorsal cortical neurons in our culture condition (Supplementary Fig. S4). For these experiments, we chose gels of two distinct stiffnesses, that is, gels prepared at [EDC 22 mM]_0.1_ (145 ± 15 Pa) and [EDC 100 mM]_0.1_ (1487 ± 62 Pa); these were defined as “SOFT” and “HARD”, respectively. HiPSCs did not grow on gels, plastic dishes or coverslips without coatings. Therefore, we tested several coating materials and chose Vitronectin XF because it provided sufficient cellular adhesion on all substrates used in this study (Supplementary Fig. S5 and Table S4). To test the possibility that the Vitronectin XF coating may change the surface structure of gels and alter the fate of stem cells, we examined the surfaces of gels coated with Vitronectin XF using confocal laser scanning microscopy and SEM (Fig. 5). Adsorbed aggregates of vitronectin were identified on both SOFT and HARD as well as coverslips used for control (hereafter “Control”) after coating (Fig. 5A,Ca,Cb). Quantification of vitronectin in the adsorption equilibrium revealed that the density of vitronectin was essentially similar between two gels and Control (Fig. 5B). In contrast, fibrous structures remained on the surface of the gels prepared by the fibril-formation method (Fig. 5Cc).

### Neural induction on tilapia collagen gels

**Figure 6:**
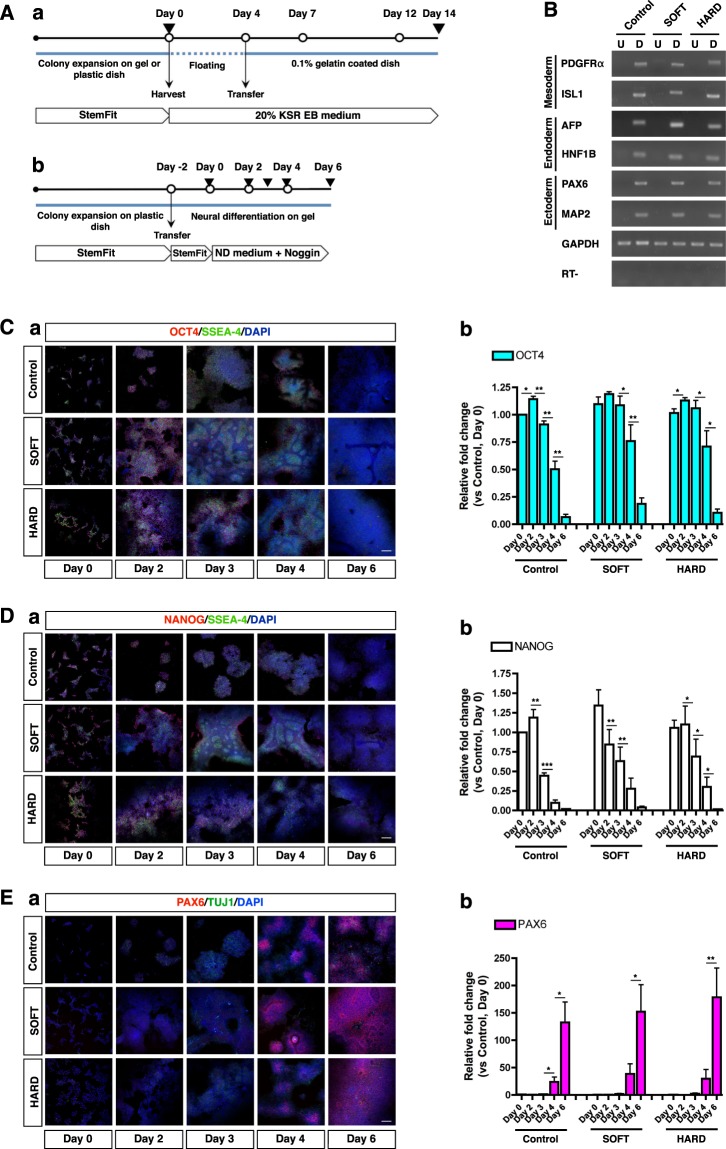
Expression of pluripotent and neural stem cell markers during neural induction on gels. (**A**) (a) Schematic of EB formation culture. White circles, the day of medium change; Black inverted triangles, the day of sampling. (b) Schematic of neural induction of hiPSCs on gels. Day 0 indicates the day on which differentiation medium was first applied. The cells were cultured on gels in plastic dishes up to Day 6. KSR EB medium, Knockout Serum Replacement EB medium; ND medium, Neural Differentiation medium; White circles, the day of medium change; Black inverted triangles, the day of sampling. (**B**) RT-PCR analysis of differentiation markers of three germ layers expressed after EB formation followed by culture on gels and plastic dishes. Mesodermal markers, PDGFRα and ISL1; Endodermal markers, AFP and HNF1B; Ectodermal markers, PAX6 and MAP2. U, undifferentiated hiPSCs on Day 0; D, differentiated hiPSCs on Day 14. (**C**) (a) Confocal images of hiPSCs stained with antibodies against OCT4 and SSEA-4. The culture conditions and the number of days after neural induction are indicated in the panel. (b) QRT-PCR analysis data showing that the mRNA level of OCT4 decreased during the early phase of culture under all conditions (n = 2). (**D**) (a) Confocal images of hiPSCs stained with antibodies against NANOG and SSEA-4. The culture conditions and the number of days after neural induction are indicated in the panel. (b) QRT-PCR analysis data showing that the mRNA level of NANOG decreased during the early phase of culture and that it was almost completely absent on Day 6 under all conditions (n = 2). (**E**) (a) Images of hiPSC-derived neural cells stained with antibodies against PAX6, TUJ1 and with DAPI. PAX6 expression was first observed on Day 4. The vast majority of cells were positive for PAX6 on Day 6 under all conditions. (b) QRT-PCR analysis of the expression of PAX6. PAX6 expression increased from Day 4 to 6. The PAX6 expression level showed no significant differences among the conditions at any of the time points tested (one-way ANOVA, n = 3). Bars = 200 μm (**C**–**E**). For the statistical analysis of qRT-PCR and counting, *P < 0.05; **P < 0.01; ***P < 0.001 (*t*-test for two consecutive days for **C** to **E**); Error bars in graph, mean ± SEM.

We aimed to examine the effect of stiffness for neural differentiation on SOFT and HARD gels and coverslips. First, we confirmed the differentiation ability of hiPSCs towards three germ layers using embryoid body (EB) assay^37^ followed by cultured on gels and coverslips (Fig. 6Aa). After transferring the EBs on dishes, cells derived from EBs showed several types of morphologies. We observed epithelial cells, cobblestone-like cells and neuronal cells under all conditions (Supplementary Fig. S6). RT-PCR analysis revealed that the expression of the three germ layer specific genes (Fig. 6B and Supplementary Fig. S7).

Next, we investigated the expression level of pluripotent markers during neural induction of hiPSCs on gels. Neural induction was initiated 2 days after passaging, which is defined as “Day 0” (Fig. 6Ab). SSEA-4, a surface marker of human pluripotent stem cells, was detected ubiquitously in hiPSCs colonies from Day 0 to 2 and disappeared on Day 6 under all conditions (Fig. 6Ca and Da). The expression of OCT4, a pluripotency marker, decreased with a similar time course (Fig. 6Ca). Quantitative RT-PCR (qRT-PCR) analysis of OCT4 mRNA supported the immunocytochemical results (Fig. 6Cb). The expression of NANOG, another pluripotency marker, gradually decreased and had disappeared on Day 6 under all conditions (Fig. 6Da). QRT-PCR analysis showed a decrease in the level of NANOG mRNA with culture progression (Fig. 6Db).

The effect of stiffness on early neural induction was investigated using PAX6 as a marker of cortical neural stem cells (Fig. 6Ea). Immunocytochemistry showed that there was no or little expression of PAX6 under any of the tested conditions on Day 0 to 3. Expression of PAX6 was detected on Day 4, and intense expression of PAX6 was observed on Day 6 under all conditions. We found TUJ1-positive cells under all conditions from Day 4 to 6; however, the population of these cells was quite small. We next performed qRT-PCR to measure PAX6 mRNA expression (Fig. 6Eb). Similar to the results obtained using immunocytochemistry, there was little expression of PAX6 under any of the tested conditions from Day 0 to 3. The level of expression of PAX6 increased rapidly from Day 4 to 6 without significant differences among the tested conditions. Both the immunostaining and the qRT-PCR results indicated that PAX6 expression showed a similar pattern in cultures maintained on gels (SOFT and HARD) and in the Control. These results imply that, regardless of the stiffness of the substrate, hiPSCs have the ability to form three germ layers and the pluripotency of hiPSCs gradually decreased and switched to neural lineage during the early stages of neural induction.

### Enhanced induction of specific neural lineages on tilapia collagen gels

**Figure 7:**
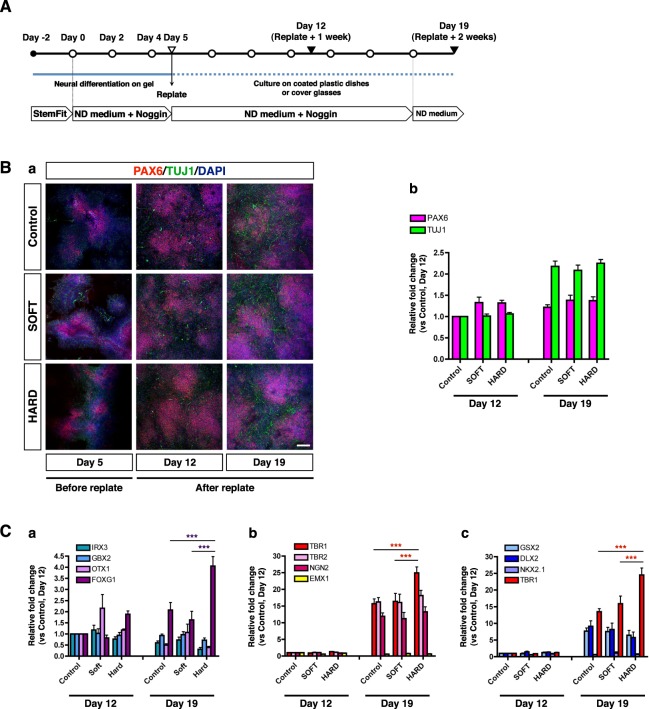
Expression of specific neural markers after neural induction. (**A**) Schematic of neural differentiation of hiPSCs that were replated after initial stiffness-stimulation. Day 0 indicates the day on which differentiation medium was applied. ND medium, Neural Differentiation medium; White circles, the day of medium change; White inverted triangle, the day of replate; Black inverted triangles, the day of sampling. (**B**) (a) Representative images of hiPSCs stained with PAX6 and TUJ1 on Day 5 (the day of replate), Day 12 (Day 5 + 1 week) and Day 19 (Day 5 + 2 weeks). (b) QRT-PCR analysis of the expression of PAX6 and TUJ1 in each culture condition. There were no significant differences under any conditions at each time point (n = 5). (**C**) QRT-PCR analysis of the expression of arealization markers in the CNS (a), dorsal cortical lineage markers (b), and ventral forebrain lineage markers (c) (n = 5). Refer to Results for details of each gene. Bars = 200 μm.

Our investigation of the initial stage of the neural induction of hiPSCs by immunocytochemistry and qRT-PCR implies that the gross differentiation states of the cells show similar tendencies irrespective of whether the cells are cultured on SOFT or HARD gels or plastic dishes. We subsequently assessed how the initial stiffness stimulation affects the differentiation ability and neural lineage determination using a replate assay^4^ (Fig. 7A). To normalize the differences in the cell numbers, we harvested attached cells from each culture condition on Day 5 and plated them on Vitronectin-coated plastic dishes at the same density, followed by culturing for up to 2 weeks. Immunocytochemistry showed that a small population of TUJ1-positive cells was observed on both types of gels and in the Control on Day 5 (the day of replate) and 1 week after replate (Day 12) (Fig. 7B). A significant increase of TUJ1-positive cells was identified 2 weeks after replate (Day 19) (Fig. 7B). QRT-PCR analysis showed that the expression levels of TUJ1 in the cells cultured on the SOFT and HARD gels were similar to that in the Control (Fig. 7Bb). Importantly, the PAX6 expression was also maintained at similar levels in all cultures on Day 19, despite the fact that neural stem cells produced more TUJ1-positive post-mitotic neurons under all conditions (Fig. 7Bb). These results indicate that hiPSCs maintained on a culture substrate with a stiffness close to that of brain tissue during the early stage of neural induction continuously generate post-mitotic neurons in a manner similar to cells cultured on control plastic dishes.

We examined a proportion of detached cells during the culture by Day 5 because this population might cause the difference in the gene expression after replate assay. We counted and summed the number of cells floating in the culture sup on Day 0 to 5. The proportions of cells and spheres that floated in the culture sup were 0.7% in SOFT, 0.5% in HARD and 0.3% in Control, which may be considered a minor population compared to the total cell number. We also addressed whether the culture on gels affects the cell survival. We found that there was no significant difference among the stiffness conditions for the CASPASE 3 expression on Day 5, Day 12 and Day 19 examined by qRT-PCR and immunocytochemistry (Supplementary Fig. S8). These results suggest that the cell survival rate is not affected by the stiffness conditions and that neural differentiation, particularly the production of progenitors and neurons, is not likely to be determined by neuronal survival.

We subsequently explored whether the initial stiffness stimulation influences the neural lineage determination by qRT-PCR (Fig. 7C). First, we tested for markers associated with arealization in the central nervous system (CNS). The expression levels of GBX2 (a diencephalic marker) and OTX1 (a marker of the forebrain and midbrain)^38^ did not show significant differences under any of the conditions. However, the expression of FOXG1, a forebrain marker^39^, significantly increased on the HARD gels, whereas that of IRX3, a marker of the midbrain and hindbrain^40,41^, was decreased in the cells cultured on the HARD gels (Fig. 7Ca). These results raise the possibility that neural progenitors and neurons induced under HARD gel conditions may have acquired a forebrain neural lineage. To further examine this possibility, we investigated the expression of dorsal and ventral forebrain markers, including markers of progenitors and post-mitotic neurons (Fig. 7Cb,Cc, respectively). The expression levels of NGN2 and TBR2 (markers of fate-committed progenitors in the dorsal cortex)^42,43^, as well as EMX1 (a marker of the dorsal telencephalon)^44,45^ were similar in all conditions (Fig. 7Cb). Notably, TBR1, a marker of newborn neurons in the dorsal cortex^43,46^, showed significantly higher expression in cells stimulated by the HARD gel (Fig. 7Cb,c). Importantly, the expression of the ventral forebrain neural lineage markers (GSX2, DLX2 and NKX2.1)^47,48^ remained at similar levels in all conditions (Fig. 7Cc). In addition, the other CNS markers, such as a GABAergic interneuron marker (GAD67)^36^, motor neuron markers (HB9 and ISL1)^49^, and an astrocyte marker (GFAP)^50^, were not significantly different among the tested conditions (Supplementary Fig. S9). These results suggest that the higher expression level of FOXG1 on HARD gels reflects an increase in the number of TBR1-positive dorsal cortical neurons rather than an increase in the number of cortical progenitors or ventral forebrain neurons.

To determine the optimal conditions to induce dorsal cortical neurons, we tested gels with intermediate stiffness, which provided approximately 780 Pa of stiffness ([EDC 50 mM]_0.1_, MIDDLE), as well as different seeding cell densities (Supplementary Figs S10 and S11). QRT-PCR showed that the expression level of TBR1 was upregulated with an increase in stiffness. The higher expression level of TBR1 on HARD was always observed irrespective of the seeding cell density. We further addressed neural differentiation from hiPSCs exposed to continuous stiffness stimulation by long-term culture on each substrate (Supplementary Fig. S12). The expression level of PAX6 was similar in both conditions, while the expressions of TUJ1 and TBR1 were significantly increased in the replate condition compared to the long-term culture. These results suggest that short-range exposure to stiffness by the replate condition is suitable to induce a dorsal neural lineage compared to the long-term culture on gels.

**Figure 8:**
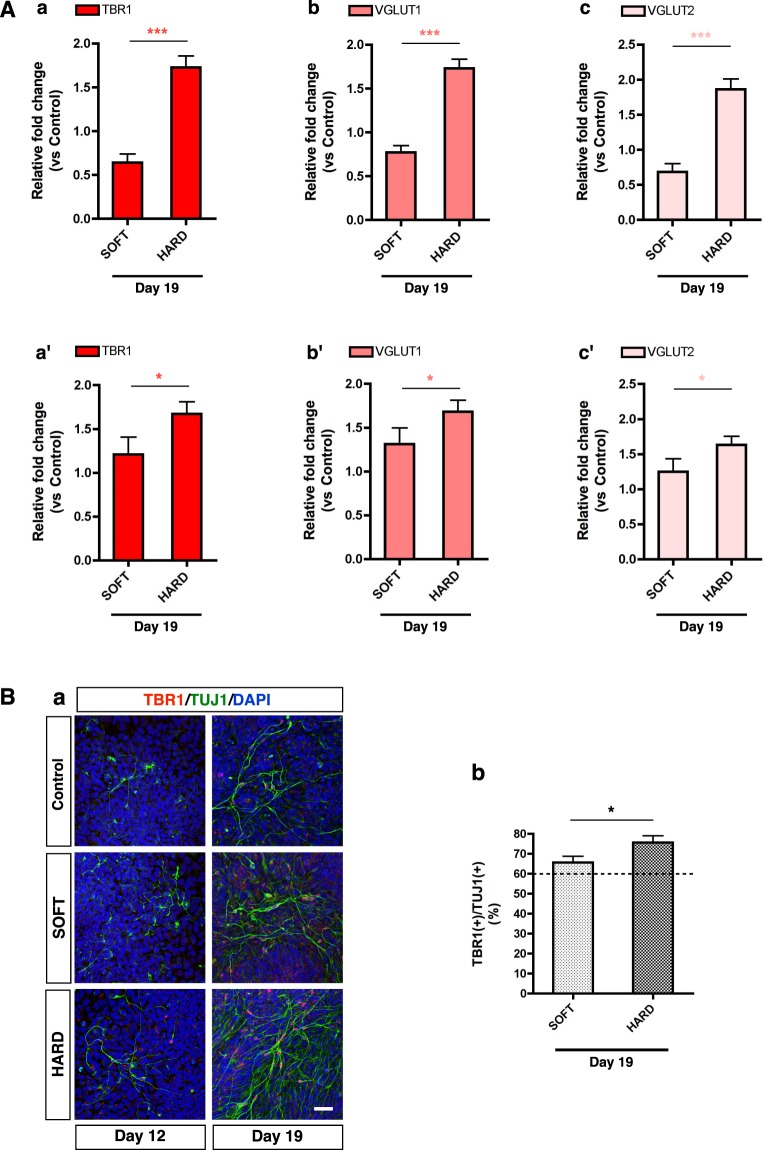
Expression of functional neuronal markers after neural differentiation. (**A**) QRT-PCR analysis of the expression of markers of glutamatergic neurons in the dorsal cortex. TBR1 is shown in (a) and (a′); VGLUT1 in (b) and (b′); and VGLUT2 in (c) and (c′). The seeded cell number on gels is 3 × 10^4^ for (a) to (c) (n = 4) and 6 × 10^4^ for (a′) to (c′) (n = 6). The gene expression level is normalized by Control on Day 19. (**B**) (a) Images of hiPSC-derived neural cells seeded at a cell density of 6 × 10^4^ stained with antibodies against TBR1 and TUJ1 and with DAPI. (b) Proportion of TBR1-positive cells among TUJ1-positive cells. Dashed line indicates the proportion of TBR1-positive neurons in Control: Control, n = 290 cells; SOFT, n = 320 cells; HARD, n = 304 cells. For statistical analysis of qRT-PCR and Proportion of cells, *P < 0.05; ***P < 0.001 (*t*-test for **A,B**); Error bar in graphs, mean ± SEM.

To confirm that TBR1-positive cells can function as neurons, we measured the expression levels of VGLUT1 and VGLUT2, markers of glutamatergic neurons in the dorsal cortex^51^, and identified significant increases in their expression under the HARD conditions (Fig. 8A). We further analyzed the TBR1 protein expression in TUJ1-positive neurons by immunocytochemistry. TBR1-positive cells were seldom observed on Day 12, but thereafter substantially increased, similar to the TUJ1-positive population under all conditions (Fig. 8Ba). Statistical analysis showed that the proportion of TBR1-positive cells among TUJ1-positive neurons was significantly increased in the cells cultured on the HARD gels (Fig. 8Bb). Taken together, stiffness stimulation due to the culture of cells on HARD gels during the early phase of neural induction (Day 0 to 5) resulted in a higher production of dorsal cortical neurons than culture on SOFT gels and culture on conventional plastic dishes.

## Discussion

In this study, we established the production of a culture substrate from tilapia collagen that is as soft as living brain tissue. To date, mammalian collagen, mostly derived from bovine and porcine skin, has been used as a biomaterial for cell culture and implantation in the human body. However, when mammalian collagen is used for these purposes, the risk of zoonoses, such as bovine spongiform encephalopathy and foot-and-mouth disease, cannot be excluded. Although PAA-based gel is an alternative biomaterial that provides a wide range of stiffness^1–6,15–18^, acrylamide displays cellular toxicity, particularly for neural cells^19^. Considering future applications in regenerative therapies, such as cell transplantation, these risks of infection and toxicity must be avoided. In addition, the procedure of PAA gel preparation is technically complex, particularly to create softer gels^52,53^. Fish collagen has been used as a biomaterial because of its low risk of pathogens and its easy acquisition from waste produced by the food and cosmetic industries^20,54,55^. Despite these advantages, the low denaturation temperature (Td) of fish collagen, which results in the dissolution of collagen fibrils under human physiological conditions, has been a limitation in its use as a biomaterial^56^. In contrast, tilapia skin collagen, likely because of its tropical origin, has a higher Td^26–28^ that is quite close to the physiological temperature of the human body. Moreover, our CD spectrum analysis (Fig. 4) showed that the specific peak indicating its triple helix structure is maintained at both 20 °C and 37 °C, which suggests that fish collagen displays structural stability under physiological culture conditions. Furthermore, the expression of collagen-related genes is not a major component in the developing brain. Accordingly, collagen might have less effect for neural cell fate determination irrespective of the stiffness stimulation compared to other extracellular matrix abundantly expressed in the brain. Therefore, we chose the skin collagen of the Nile tilapia as a material for culture substrate.

Although collagen gels with a higher stiffness have previously been reported^57,58^, the brain is one of the softest tissues in the body^7–9^. To generate a “soft” substrate with a stiffness similar to that of brain tissue, we investigated several conditions for the use of NHS and EDC. We demonstrated that a ratio of NHS to EDC ([NHS]/[EDC]) = 0.1 is suitable for producing gels with a stiffness of 150–1500 Pa with high reproducibility. This range of stiffness is similar to the range of stiffness measured in the developing^13,14^ and adult brain^11,12^. This result suggests that the stiffness of living brain tissue can be reproduced *in vitro* using tilapia collagen. In contrast, this range of stiffness would not be suitable for the culture of other cell types, which naturally grow under much stiffer conditions such as those found in muscle and bone^1,2^. However, we do not exclude the possibility of obtaining gels of greater stiffness by changing the concentrations of collagen and crosslinking reagents.

SDS-PAGE indicated that collagen isolated from tilapia skin by acid extraction consists of α1, α2, and β chains, similar to commercially available collagens (Fig. 1A and Supplementary Fig. S1A). In addition, the fish specific collage type I α3 was identified via MALDI-TOF/MS analysis (Fig. 1B and Supplementary Table S1). The molecular weight of each component falls within the previously reported range^26–28^. These results suggest that tilapia skin collagen can be used for gel formation in a manner similar to mammalian collagen. In general, monomeric collagen extracted from various species forms self-assembling fibrils under physiological conditions. Chemical modification promotes the interaction between monomeric collagen fibers during self-assembly^33^. Furthermore, the triple helical structure was maintained in crosslinked tilapia collagen gels (Fig. 4C and D). The triple helix in collagen is known to have a GFOGER sequence on its surface, and this sequence interacts with α1, α1β1, α2β1, α10β1 and α11β1 integrins on the cell surface to act as a mediator of mechanical stimulation, including stiffness^59^. Thus, it is possible that the stiffness stimulation of cells grown on crosslinked tilapia collagen gels is directly transmitted via integrin.

Using chemically crosslinked tilapia collagen gels, we confirmed that the loss of pluripotency and induction of gross neural lineages from hiPSCs are promoted by the culture of hiPSCs on gels in a similar manner to hiPSCs grown on conventional substrates (plastic dishes and coverslips). Previous studies using PAA gels and poly micropost arrays indicated that the production of PAX6- and TUJ1-positive cells from human pluripotent stem cells was enhanced under softer gel conditions (0.1 to 5 kPa)^4–6^. The difference in the origin of the gels, as well as other aspects of the methodology, such as the coating substrate used and the ingredients in the neural induction medium, might be the cause of the differences. Surprisingly, we found that stiffness stimulation in the earlier phase of neural induction enhances the production of neurons typical of those found in the dorsal cortex (Fig. 7). Intriguingly, the expression of TUJ1 was similar in cells cultured on gels and control plastic dishes, whereas the population of TBR1-positive neurons significantly increased compared to the conventional substrate at 2 weeks after replate. Furthermore, markers of glutamatergic neurons in the dorsal cortex (VGLUT1 and 2) were also upregulated (Fig. 8). Remarkably, the expression levels of markers for cells in other CNS regions, including ventral forebrain and dorsal neural progenitors were essentially similar under all culture conditions (Fig. 7C). Considering that the medium conditions^36^ and hiPS cell-lines (Supplementary Fig. S4) were set to favor the production of dorsal cortical neurons, stiffness stimulation in the early phase of neural induction may have additional important effects as an insoluble mechanical factor for the determination of the terminal neural subtype^4–6^. The enhanced production of dorsal cortical neurons from hiPSCs on gels could be useful in neural regenerative studies, including transplantation to animal models, as well as in the treatment of cortical dysfunctions and drug screening.

Chemically crosslinked tilapia collagen gels have strong advantages due to their appearance. The kinetics of the *G*′ analysis during crosslinking indicated that tilapia collagen gels exhibit typical properties of elastic gels. Furthermore, SEM analysis indicated that crosslinked tilapia collagen gels have a topographical homogeneity of their surface regardless of the difference in stiffness (Fig. 1D). Previous studies suggest that the surface of the culture substrate, such as the nanostructure, fiber diameter and topography^30–32^ and peptide sequence^60^, may affect the cell fate determination. The flattened structure of chemically crosslinked gels excludes the possibility of topographical effects and supports the homogeneous attachment of ligand on its surface. In addition, crosslinked tilapia collagen gels showed high transparency (Fig. 1C and Supplementary Fig. S1C), which provides a substantial advantage for visualizing cellular and molecular behaviors on gels combined with live microscopy. In summary, our results demonstrate that chemically crosslinked tilapia collagen gel can provide stiffness that mimics brain tissue with the advantages of high reproducibility, as well as fewer possibilities of zoonosis and neural toxicity, which are desirable features to culture substrates for various studies of neural lineages and functional analyses, as well as for regenerative therapeutic applications.

## Methods

All methods were carried out in accordance with the Experimental Guidelines and Regulations in Korea Brain Research Institute. All the experimental protocols were approved by the Committee in Korea Brain Research Institute.

### Collagen extraction from fish skin

Tilapia collagen was extracted from the frozen skin of the animals according to a previously reported procedure^26–28^ with some modifications. All procedures were performed in a cold room. Defrosted tilapia skin was defatted in three steps. First, the skin was washed with a solution of 30% chloroform/64% methanol for 10 min and then washed with methanol for 10 min. The skin was then washed twice with 100 volumes of distilled water (DW) for 1 h. Subsequently, acid extraction was performed by soaking the tilapia skin in 3% CH_3_COOH for 3 days. The collagen solution was gently filtered through gauze and filtered 4 times over a 1-μm-pore filter (TCPD-05A-D1HS 3 times and TCPD-02-D1HS once, Advantec). Salting-out and acid solubilization of the collagen were performed twice using 0.7 M saturated brine. For this, 0.7 M saturated brine was gently added to the filtered product, and the mixture was incubated for 4 h. After draining the water overnight, the products were cut into small pieces and soaked in 1.2% CH_3_COOH. The acidic mixture was stirred for 30 min. After salting-out twice, dialysis was performed. The extracted product was dissolved in 0.01% CH_3_COOH, and the collagen solution was placed in a dialysis membrane (UC36-32-500, Sekisui Chemical). Upon reaching pH 5.0, the inner liquid was replaced with HCl (pH 3.0). The product was sequentially filtered three times using 1-μm (Y100A090A, Advantec), 0.65-μm (A065A090C, Advantec) and 0.45-μm (C045A090A, Advantec) membrane filters. The final product was aliquoted and stored at −20 °C. To confirm the protein sequence of extracted collagen, MALDI-TOF/MS was performed. Details are described in Supplementary methods.

### SDS-PAGE

Tilapia skin collagen was diluted into HCl solution (pH 3.0). To compare the components of collagen, similar amounts of porcine tendon (Nitta gelatin) and tilapia scale collagen (Taki Kagaku) were loaded on the same gels. Details are described in Supplementary methods.

### Preparation of collagen gels using crosslinking and fibril-formation methods

To obtain gels of various stiffnesses, 1-ethyl-3-(3-dimethylaminopropyl)-carbodiimide (EDC) (Dojindo), N-hydroxysuccinimide (NHS) (Wako) and NaCl solution (Sigma-Aldrich) were diluted into DW at various combinations of final concentrations as shown in Supplementary Tables S2 and S3. The defrosted stock collagen solution and the crosslinking solution were kept on ice before mixing. The crosslinking solution was added to the collagen solution in a 50-ml tube, and the mixture was shaken 20 times by hand. The tube was then centrifuged at 1000 rpm at 4 °C for 1 min. After centrifugation, the tube was immediately placed on ice. Two and five milliliters of gel solution was poured into 35-mm and 60-mm dishes, respectively. The gels were stored at 25 °C overnight and washed with PBS 6 times for 40 min each time. The final concentration of collagen in the gels was 0.3 wt%.

For fibril formation, all procedures were performed on ice according to the manufacturer’s instructions (Nitta Gelatin). The tilapia collagen solution was mixed well with 10x concentrated DMEM/F12 (Sigma-Aldrich) by pipetting. Reconstruction buffer containing 50 mM sodium hydroxide, 260 mM sodium hydrogen carbonate and 200 mM HEPES was then added, and the components were gently mixed by pipetting. The ratio of components was 8:1:1 (collagen solution, 10x DMEM/F12 and reconstruction buffer, respectively). The final concentration of collagen was 0.24 wt%.

### Mechanical characterization of materials

The bulk stiffness of the materials was measured using a rheometer (Thermo Fisher Scientific). Measurements were conducted using a parallel plate (35 mm diameter) at 37 °C for consistency with the cell culture conditions. The frequency-dependent storage and loss modulus were measured by applying 1 N of force 24 h after crosslinking. The time-dependent storage and loss modulus were measured immediately after mixing the collagen solution and crosslinker for 1 h. The values of the gap from the bottom of the dish at 1 N of force were defined as the “height” of the gel.

### Measurement of crosslinking degree and turbidity

To evaluate the degree of crosslinking of the collagen, the free amine group content was measured following previous procedures^22^. First, the crosslinked collagen gels were freeze-dried. Four milligrams of freeze-dried gel was collected in a glass bottle, and 1 ml of NaHCO_3_ solution (4% w/v) and 0.5% TNBS solution (Thermo Fisher Scientific) was then added. The glass bottle was incubated in a water bath at 40 °C for 2 h. Then, 3 ml of 6 N HCl was added. The temperature of the water bath was increased to 60 °C, and the glass bottle was incubated for an additional 90 min. After the gel had dissolved completely, the reaction solution was diluted with 5 ml of DW, and 100 μl of the reaction solution was placed in each well of a 96-well plate. The absorbance of the solution at 345 nm was measured at room temperature (25 °C) using a photometer (Tecan). Collagen solution without crosslinking was used as a control.

To quantitate the turbidity of gels during crosslinking, the absorbance of the sample at 310 nm was measured using a photometer (Molecular Devices). Gels were prepared using the crosslinking and fibril-formation methods as described above. One hundred microliters of gel solution was placed in the well of a 96-well plate, and the plate was kept on ice prior to measurement. Measurement of turbidity was performed at room temperature. Turbidity was monitored continuously for 3600 sec at 30-sec intervals.

### Measurement of CD spectrum

To determine whether the native triple helix of collagen was present in the gel samples, the circular dichroism (CD) spectra of fibril-formed physical collagen gels and chemically crosslinked collagen gels were measured. Because the gel samples were generally too concentrated to obtain clear CD spectra due to their strong absorption, the measurement was performed under conditions of reduced optical path length using a 30-μm-thick spacer between two pieces of quartz glass plate. For the physical gel sample, 20 μl of an aqueous solution of collagen (0.3 wt%, pH 3.0) was placed between the two quartz glass plates. For the crosslinked gel sample, the collagen solution, EDC, and NHS were first mixed and placed between a standard cover glass and the quartz glass plates. The cover glass was pre-coated with polyethylene glycol (PEG, Mw 500,000; Sigma-Aldrich)^61^. The PEG-coated cover glass was then removed in PBS after the crosslinking reaction, and the formed collagen gels were washed thoroughly with PBS to remove the remaining crosslinking reagents. Finally, the collagen gels prepared on the quartz glass were covered with another quartz glass plate using a 30-μm-thick spacer (ESCO Co., Ltd.). The collagen gel sample sandwiched between two pieces of quartz glass was placed in the optical path of a CD spectrometer (J-720W, JASCO Corp.), and the CD spectrum of the sample was measured.

### Culture of human iPSCs on gels

Use of human-derived material in the research is approved by the Research Ethics Committee of Korea Brain Research Institute (KBRI-201603-BR-001-01). Four different hiPSCs lines were tested to characterize the variance in the neural induction. Among them, 1231A3, 1383D2, and 1383D6 (obtained from Center for iPS Cell Research and Application, Kyoto Univ, Japan) were established by the reprogramming method using episomal plasmids^62^, while RPChiPS771-2 (obtained from ReproCELL, Japan) were established by the self-replicative RNA reprogramming method^63^. Expansion and maintenance of hiPSCs were performed according to previously reported feeder-free culture methods for human pluripotent stem cells^62^ except that Vitronectin XF (10 µg/ml final concentration, StemCell Technologies) was used for coating. Neural differentiation was performed as described previously^36^ with some modifications. Two days prior to differentiation, the cells were dissociated using 0.5x TrypLE Select (1x TrypLE Select, Life Technologies, diluted 1:1 with 0.5 mM EDTA/PBS(−)) and seeded at a density of 1× , 3× , or 6 × 10^4^ cells per 35-mm dish on Vitronectin XF-coated gels or on glass coverslips in StemFit Basic02 medium (Ajinomoto). On day 0, the medium was replaced with recombinant human Noggin (R&D systems)-containing neural differentiation medium^36^. A medium change was performed every 2 days. For replate experiments, the cells were dissociated with 0.25% trypsin-EDTA on day 5 and replated on Vitronectin XF-coated 35-mm plastic dishes at a density of 2 × 10^5^ cells per dish. After replate, the cells were cultured in Noggin-containing differentiation medium for 10 days; the medium was then changed to differentiation medium without Noggin for 4 days. Embryoid body (EB) formation assay^37^ is described in Supplementary methods.

### Immunocytochemistry and quantitative analysis

To measure the neural differentiation of hiPSCs, immunocytochemistry was performed. The fixed cells were incubated with primary antibodies and then with secondary antibodies. The information of antibodies is shown in Supplementary Table S5. Immunofluorescence images were acquired using a Nikon A1R-MP confocal laser scanning microscope (Nikon). To investigate the distribution of ligand on culture substrate, vitronectin-coated area was quantified using confocal images. Detail is described in Supplementary methods.

### Quantitative RT-PCR (qRT-PCR)

To determine the gene expression levels of hiPSCs cultured on gels, qRT-PCR was performed. Triplicate reactions were run for each biological sample using ABI7500fast (Life Technologies). The primer sequences are listed in Supplementary Table S6. Relative fold changes in mRNA expression were calculated using the 2^−∆∆Ct^ method. Details are shown in Supplementary methods.

### Statistical analysis

Statistical analysis was performed using Prism version 4.0 (Graphpad Software). Two-tailed paired *t*-test was applied to compare two conditions (Control vs SOFT, Control vs HARD and SOFT vs HARD) and one-way ANOVA and Tukey *post hoc* test was applied to compare three conditions at the same time points. Differences were considered significant for *P* < 0.05 (*), *P* < 0.01 (**), *P* < 0.001 (***). Error bars in graphs were represented as the mean ± SEM.

## Supplementary information

Supplementary Information
