# Supplementary material for: Brain-stiffness-mimicking tilapia collagen gel promotes the induction of dorsal cortical neurons from human pluripotent stem cells
Source: Sci Rep. 2019 Feb 28;9:3068. doi: 10.1038/s41598-018-38395-5 (PMC6395773; doi:10.1038/s41598-018-38395-5)
Supplement: Supplementary file 1 — Supplementary Information [file 41598_2018_38395_MOESM1_ESM.pdf]

Supplementary Information

Brain-stiffness-mimicking tilapia collagen gel promotes the induction of dorsal cortical neurons from human pluripotent stem cells

Authors

Misato Iwashita[a], Hatsumi Ohta[b], Takahiro Fujisawa[c], Minyoung Cho[a], Makoto Ikeya[d], Satoru Kidoaki[c] and Yoichi Kosodo[a, *]

Affiliations

[a] Korea Brain Research Institute, 61, Chemdan-ro, Dong-gu, Daegu, 41068, Republic of Korea

[b] Ihara & Co, Ltd, 3-263-23, Zenibako, Otaru, Hokkaido 947-0261, Japan

[c] Institute for Materials Chemistry and Engineering, Kyushu University, 744 Motooka, Nishi-ku, Fukuoka 819-0395, Japan

[d] Center for iPS Cell Research and Application (CiRA), Kyoto University, 53 Kawahara-cho, Shogoin, Sakyo-ku, Kyoto 606-8507, Japan

* Corresponding author (kosodo@kbri.re.kr)

Supplementary Methods

SDS-PAGE

The protein concentration of the solution was measured using Qubit Protein Assay Kits (Q33211, Life Technologies). Five micrograms of protein were used for SDS-PAGE. The collagen solution was mixed with 2x sample buffer containing 2-mercaptoethanol (161-0737, Bio-Rad Laboratories) and denatured at 98˚C for 3 min. SDS-PAGE was performed using Tris-HCl/glycine/SDS-based buffer (161-0772, Bio-Rad Laboratories) and Any kD precast gels (456-9033, Bio-Rad Laboratories). Molecular weight markers ranging in size from 10 to 250 kDa (Precision Plus Protein Standards, 161-0373, Bio-Rad Laboratories) were loaded in lanes on both sides of the lanes containing the collagen samples. Electrophoresis was performed using a PowerPac (HC High-Current Power Supply, Bio-Rad Laboratories) at a constant current of 20 mA at room temperature for 1 h. After electrophoresis, the gel was stained with pre-made Coomassie blue solution (SimplyBlue SafeStain, LC6060, Life Technologies) according to the manufacturer’s instructions.

Peptide sequence of collagen derived from tilapia skin

Three bands were manually cut after SDS-PAGE (Supplementary Fig. S1Bb). Proteins included in each band were subsequently digested by treatment with Trypsin. MALDI-TOF/MS analysis was performed using a Microflex LRF 20 (Bruker) (GENOMINE Inc., Pohang, Korea). The obtained peptide sequences are listed in Supplementary Table S1. Each peptide sequence was aligned using Clustal Omega (version 1.2.4).

Scanning electron microscopy (SEM) of collagen gels

The surface structure of gels was observed using an SEM (SU8220, Hitachi). The fixation and drying procedures were as follows. The gels were fixed with 2.5% glutaraldehyde (25% stock, 18427, Ted Pella) in 0.1 M phosphate buffer (PB) at 4˚C overnight. After 2 washes in 0.1 M PB for 15 min, the gels were post-fixed with 1% OsO_4 (2% aqueous stock, 18466, Ted Pella) at 4˚C for 1 h, then washed twice in 0.1 M PB for 15 min. Subsequently, the gels were dehydrated by sequential immersion in 50, 60, 70, 80, 90 and 95% ethanol for 10 min at 4˚C. The gels were then dehydrated by immersion in 100% ethanol for 15 min twice at 4˚C. Before freezing the gels, the ethanol was replaced with t-butyl alcohol by incubating the gels in t-butyl alcohol twice for 20 min at room temperature. Finally, the gels were soaked in t-butyl alcohol, placed at 4˚C overnight, and

freeze-dried using a vacuum freeze dryer (IlShin Valves). Samples were affixed to the stage with carbon tape, and vapor deposition with platinum (E-1030, Hitachi) was performed prior to observation.

Quantitative analysis of ligand distribution

To investigate the distribution of vitronectin on the culture substrate, gels (SOFT and HARD) and coverslips were coated with Vitronectin XF as described in the Methods. The coated culture substrates were subsequently fixed with 4% PFA. Vitronectin was detected by anti-vitronectin antibody and visualized by Alexa488 similar to immunocytochemistry. The surface images of the culture substrate were subsequently acquired using a confocal microscope (Nikon A1R-MP). To obtain a higher magnified view, a 60x oil objective lens with 5x digital zoom was used. Acquired images were analyzed by ImageJ. The background was initially subtracted, and the vitronectin-positive region was then extracted using the threshold and converted to the binary image. Finally, the extracted area was measured. Statistical analysis was performed using Prism version 4.0 (GraphPad Software). The antibody information is described in Supplementary Table S5.

Immunocytochemistry and microscopy of cultured cells

The cells were fixed in medium containing 4% PFA at room temperature for 10 min. After washing with PBS 3 times for 5 min each, the cells were permeabilized with 0.5% Triton X-100 in PBS for 10 min. The cells were then washed with PBS 3 times for 5 min each and blocked with 2% BSA in PBS containing 0.1% Triton X-100 at room temperature for 1 h. The cells were incubated with primary antibodies at 4˚C overnight and then with secondary antibodies at room temperature for 2 h. Counterstaining was performed using DAPI (Sigma-Aldrich). The information of antibodies is described in Supplementary Table S5.

Quantitative RT-PCR (qRT-PCR)

Total RNA was extracted using an RNeasy Mini Kit (Qiagen) according to the manufacturer’s instructions. cDNA was synthesized using the SuperScript VILO cDNA Synthesis Kit (Life Technologies). Five nanograms of cDNA were mixed with FastStart Universal SYBR Green Master (ROX) (Roche) according to the manufacturer’s instructions. Triplicate reactions were run for each biological sample using ABI7500fast (Life Technologies). The primer sequences for qRT-PCR are listed in Supplementary Table S6 and are derived from previously published sequences. Relative

fold changes in mRNA expression were calculated using the $2^{-\Delta\Delta Ct}$ method: $2^{-\Delta\Delta Ct}$ in which $\Delta Ct = Ct_{target} - Ct_{normalizer}$, $\Delta\Delta Ct = \Delta Ct - \Delta Ct_{reference}$. GAPDH, a housekeeping gene, was used to normalize gene expression. In the experiment shown in Fig. 6C to E, the value of ΔCt on Day 0 was used as a reference in each condition. In the experiments shown in Fig. 7B and C, the value of Controls on Day 12 was used as references. Controls on Day 19 were used as references in the experiments shown in Fig. 8A and Supplementary Figs. S9, 10, and 11. In the Supplementary Fig. S4A and B, 1231A3 on Day 12 and on Day 19 were used as reference, respectively. In the Supplementary Fig. S8B, Control on Day 5 was used as a reference. For Supplementary Fig. S12, Control on Day 19 in long-term culture condition was used as a reference.

Embryoid body (EB) formation

Human iPS cells were expanded on gels or plastic dishes coated with Vitronectin in StemFit medium. After 1 week, the hiPSC colonies were detached by treatment with Dispase I (0.5 Unit/ml) (BD Biosciences). Harvested cells were transferred to a petri dish in DMEM/F12/GlutaMAX-I (Invitrogen) that contained 20% knockout serum replacement (Invitrogen) (20% KSR EB medium), 0.1 mM of Non-Essential Amino Acids (Invitrogen), 0.1 mM of 2-mercaptoethanol (Invitrogen) and penicillin/streptomycin (Invitrogen). After 4 days of culture in the floating condition, the EBs

were transferred to a 0.1% gelatin-coated dish and cultured in the same medium for an additional 10 days. The medium was changed on Day 4, 7 and 12. EBs were collected for RNA extraction with DNase I treatment on Day 14. cDNA was synthesized as described above. To confirm the undifferentiated state, RNA was extracted from expanded hiPSCs on the harvested day. RT-PCR was performed to confirm the gene expression of three germ layer markers. The primer sequences for RT-PCR are listed in Supplementary Table S7.

Supplementary Figure Legends

Supplementary Figure S1. Original gel images of SDS-PAGE and gel appearance in Figure 1

(A) The original gel image of SDS-PAGE in Figure 1A.

(B) **(a)** The original gel image of SDS-PAGE for MALDI-TOF/MS analysis. **(b)** The gel image after cutting the bands of collagen β1 (No.1), α1 (No.2), and α2 (No.3) in **(a)**.

(C) The original images of collagen gel appearance in Figure 1C.

Supplementary Figure S2. The *G'* and *G"* value in each EDC concentration condition in Figure 2C

The graph of *G'* and *G"* in Figure 2C was divided into each EDC concentration condition, **(A)** 10 mM, **(B)** 20 mM, **(C)** 22 mM, **(D)** 50 mM, **(E)** 100 mM, **(F)** 150 mM and **(G)** 200 mM of EDC. Solid and dashed lines indicate *G'* and *G"*, respectively.

Supplementary Figure S3. The kinetics of *G'* and *G"* during crosslinking formation

Typical time plot of kinetics of *G'* and *G"*. **(A)** 22 mM, **(B)** 100 mM of EDC. Solid and dashed lines indicate *G'* and *G"*, respectively.

Supplementary Figure S4. Variance in the production rate of dorsal cortical neurons among hiPS cell-lines

Gene expression analysis in four hiPS cell-lines using post-mitotic neuron marker (TUJ1, **a**), dorsal progenitor marker (TBR2, **b**), and dorsal neuron marker (TBR1, **c**) by qRT-PCR. Results of two independent experiments are shown as **(A)** and **(B)**. QRT-PCR was run using triplicate for each cell line. Notably, the highest expressions of TBR2 and TBR1 were observed in RPChiPS771-2 in both **(A)** and **(B)**. Error bar in graphs, mean ± SEM.

Supplementary Figure S5. Optimization of coating conditions for gels

HiPSCs were cultured in maintenance medium (StemFit) containing basic FGF for 6 days on tilapia collagen gel with various kinds of coating materials (shown in Supplementary Table S4). HiPSCs cultured on gels coated with Vitronectin XF **(D)**, Matrigel **(E)**, Geltrex **(F)** or Vitronectin N **(H)** spread over the entire gel surface, whereas cells cultured on Poly-L-ornithine (PLO) plus fibronectin **(C)** or laminin **(G)** formed small colonies. HiPSCs cultured on gels lacking a coating **(A)** or on gels coated with iMatrix **(B)** hardly attached to the surface. Bar = 500 μm.

Supplementary Figure S6. Embryoid body (EB) mediated differentiation of hiPSCs exposed to stiffness stimulation

Representative images of EBs on Day 4 (**a**, **f** and **k**) and differentiated cells on Day 14 (**b**, **c**, **d** and **e** in Control; **g**, **h**, **i** and **j** in SOFT; and **l**, **m**, **n** and **o** in HARD). Harvested hiPSCs formed EBs (**a**, **f** and **k**). After transfer of EBs to gelatin-coated dishes, cells expanded from EB (**b**, **g** and **l**) and showed distinctive morphologies. Epithelial-like cells (arrows in **c**, **h** and **m**), cobblestone-like cells (arrowheads in **d**, **i** and **n**) and neuron-like cells (triangles in **e**, **j** and **o**) were observed. Bars = 200 μm (**a**, **c**, **f**, **h**, **k** and **m**) and 500 μm (**b**, **g** and **i**).

Supplementary Figure S7. Original gel images of RT-PCR

(A) PDGFRα , **(B)** ISL1, **(C)** AFP, **(D)** HNF1B, **(E)** PAX6, **(F)** MAP2, **(G)** GAPDH, **(H)** GAPDH in RT(-). U, undifferentiated hiPSCs harvested on Day 0; D, differentiated hiPSCs, harvested on Day 14.

Supplementary Figure S8. The effect of apoptosis on neural differentiation

(A) Representative images of CASPASE3 immunocytochemistry on Day 5 and 19.

(B) Relative expression level of CASPASE3 on Day 5, 12 and 19 acquired by qRT-PCR. There is

no significant difference in any combination (one-way ANOVA).

Supplementary Figure S9. QRT-PCR analysis of representative markers expressed in the central nervous system.

(A) GAD67, GABAergic interneurons; **(B)** HB9, motor neurons in spinal cord; **(C)** ISL1, motor neurons in spinal cord; **(D)** GFAP, astrocytes.

There is no significant difference in any combination (one-way ANOVA). (n = 5)

Supplementary Figure S10. Comparison of gene expression pattern in different cell densities

(A) Gene expression patterns of representative markers (PAX6 **(a)**, TUJ1 **(b)** and TBR1 **(c)**) on Day 19 acquired by qRT-PCR at a density of $1x10^4$ cells seeded per dish.

(B) Gene expression patterns of representative markers (PAX6 **(a)**, TUJ1 **(b)** and TBR1 **(c)**) on Day 19 acquired by qRT-PCR at a density of $3x10^4$ cells seeded per dish.

For statistical analysis of qRT-PCR, *, $P < 0.05$; **, $P < 0.01$; ***, $P < 0.001$ (one-way ANOVA and Turkey *post hoc* test for **A** and **B**); Error bar in graphs, mean ± SEM. (n = 4 for each condition)

Supplementary Figure S11. Comparison of gene expression patterns in various stiffness conditions

HiPSCs were cultured on SOFT (150 Pa), MIDDLE (780 Pa), HARD (1500 Pa) and Control conditions using the replate method.

(A) Gene expression patterns of representative markers (PAX6 **(a)**, TUJ1 **(b)** and TBR1 **(c)**) on Day 19 acquired by qRT-PCR with various stiffness at a density of $1x10^4$ cells seeded per dish.

(B) Gene expression patterns of representative markers (PAX6 **(a)**, TUJ1 **(b)** and TBR1 **(c)**) on Day 19 acquired by qRT-PCR with various stiffness at a density of $3x10^4$ cells seeded per dish.

For statistical analysis of qRT-PCR, *, $P < 0.05$; **, $P < 0.01$; ***, $P < 0.001$ (one-way ANOVA and Turkey *post hoc* test for **A** and **B**); Error bar in graphs, mean ± SEM. (n = 2 for each condition)

Supplementary Figure S12. Long−term neural differentiation on gels without replate

(A) Representative images of cultured hiPSCs during 19-day culture on gels and plates. We observed that some hiPSC colonies tended to show three-dimensional growth in the vertical direction and formed a sphere-like structure particularly on gels. These spheres grew during culture, and some spheres detached from the colony. Detached spheres eventually re-attached to the gel surface and started to grow again. We compared the representative gene expressions

in neural differentiation between long-term culture and replate conditions **(B)**. Bar = 500 μm.

(B) Gene expression pattern of representative neural differentiation markers, PAX6 **(a)**, TUJ1 **(b)** and TBR1 **(c)** on Day 19. For statistical analysis of qRT-PCR, *, $P < 0.05$; **, $P < 0.01$; ***, $P < 0.001$ (one-way ANOVA and Turkey *post hoc* test for **B**); Error bar in graphs, mean ± SEM. (n = 3 for long-term, n = 5 for replate)

Supplementary Table S1. Detected sequences by MALDI-TOF/MS

Band No.	Sequence No.	Identified peptide	Protein view	Taxonomy
No.1	Sequence_1	R.DQKGLGFGPIK.R	gi|551524684	*Xiphophorus maculatus*
	Sequence_2	K.GSPGAAGISGAPGFPGSR.G	gi|208609649	*Carassius auratus*
	Sequence_3	R.LRAAVAGEEAGGGKEK.K	gi|551517022	*Xiphophorus maculatus*
	Sequence_4	R.IRAGKNLCYYER.V	gi|542219479	*Oreochromis niloticus*
	Sequence_5	K.DGQIKGVTVDK.A	gi|584021909	*Neolamprologus brichardi*
No.2	Sequence_2	K.GSPGAAGISGAPGFPGSR.G	gi|208609649	*Carassius auratus*
	Sequence_6	K.GPKPEACGAAPMKR.H	gi|642077679	*Oncorhynchus mykiss*
	Sequence_7	R.GLPGPAGLPGVAGPPGR.T	gi|642072031	*Oncorhynchus mykiss*
	Sequence_8	K.QAQNIITLCAAVPR.K	gi|499034696	*Maylandia zebra*
	Sequence_9	R.AKGLQLASSKEIAAR.A	gi|642036541	*Oncorhynchus mykiss*
No.3	Sequence_1	R.DQKGLGFGPIK.R	gi|551524684	*Xiphophorus maculatus*
	Sequence_10	R.QQREQQGQPGYTMQR.S + Oxidation (M)	gi|657589372	*Stegastes partitus*
	Sequence_11	K.NIVSKGGSRNK.V	gi|642131988	*Oncorhynchus mykiss*
	Sequence_12	R.IIGMLSPDELGLFR.E + Oxidation (M)	gi|657737823	*Cynoglossus semilaevis*
	Sequence_13	R.LIGSNQETLGIMER.F + Oxidation (M)	gi|432889040	*Oryzias latipes*

Supplementary Table S2. Effect of the [NHS]/[EDC] ratio on gel stiffness

Ratio	EDC (mM)	NHS (mM)	*G'* (Pa)
0	20	0	67 ± 3
	50	0	290 ± 13
	100	0	496 ± 6
0.001	20	0.02	72 ± 6
	50	0.05	221 ± 37
	100	0.1	424 ± 24
0.01	20	0.2	56 ± 4
	50	0.5	158 ± 5
	100	1	1126 ± 42
0.1	20	2	72 ± 6
	50	5	1017 ± 63
	100	10	1682 ± 69
0.5	20	10	438 ± 36
	50	25	1080 ± 15
	100	50	1565 ± 72
1	20	20	297 ± 25
	50	50	529 ± 59
	100	100	430 ± 2

(mean ± SEM for *G'*)

Supplementary Table S3. Combinations of reagents used in the crosslinking method

EDC (mM)	NHS (mM)	NaCl (mM)	Collagen (%)	*G'* (Pa)	*G"* (Pa)	Thickness (mm)
200	20	50	0.3	1663 ± 62	71 ± 14	1.623 ± 0.05
150	15	50	0.3	1654 ± 27	65 ± 29	1.609 ± 0.03
100	10	50	0.3	1487 ± 62	134 ± 15	1.630 ± 0.02
50	5	50	0.3	780 ± 17	73 ± 5	1.632 ± 0.06
22	2.2	50	0.3	145 ± 15	33 ± 2	1.498 ± 0.04
20	2	50	0.3	136 ± 13	30± 1	1.483 ± 0.06
10	1	50	0.3	13 ± 7	4 ± 1	0.568 ± 0.1

(mean ± SEM for *G', G"* and thickness)

Supplementary Table S4. List of coating reagents

Coating solution	Supplier	Catalog No.	Final conc. (1 ml/35 mm dish)	Coating condition
Matrigel	Corning	354277	10 µg/ml	1 h
Geltrex (x100)	Gibco	A1413201	x1	1 h
Vitronectin XF	StemCell Technologies	#07180	10 µg/ml	1 h
Vitronectin	ThermoFisher	VTN-N #A14700	5 µg/ml	1 h
iMatrix-511	Takara	#892011	6.4 µl/ml	1 h
Polyornithine<br>Laminin	Sigma-Aldrich<br>Wako	P3655<br>120-05751	15 µg/ml<br>1 µg/ml	Poly-L-ornithine O/N, then laminin for 6 h
Polyornithine<br>Fibronectin	Sigma-Aldrich<br>R&D Systems	P3655<br>1030-FN	15 µg/ml<br>1 µg/ml	Poly-L-ornithine O/N, then fibronectin for 6h

Supplementary Table S5. List of antibodies

Primary antibodies	Species	Company	catalog Number	Dilution
Pax6	Rabbit	MBL	PD022	1:1000
Tbr1	Rabbit	Abcam	ab31490	1:1000
Oct4	Rabbit	Abcam	ab19857	1:1000
Nanog	Rabbit	MBL	PM058	1:1000
Tuj1	Mouse	Covance	MMS-435P	1:1000
SSEA-4 (813-70)	Mouse	Santa Cruz	sc-21704	1:1000
Vitronectin (vn58-1)	Mouse	Abcam	ab13413	1:2000

Secondary antibodies	Species	Company	catalog Number	Dilution
anti-rabbit Alexa 555	goat	Life Technologies	A21428	1:1000
anti-mouse Alexa 488	goat	Life Technologies	A11001	1:1000

Supplementary Table S6. Primer sequences used in qRT-PCR

Gene	Primer	Sequence	Reference
NANOG	Forward	CAGTCTGGACACTGGCTGAA	1
	Reverse	CTCGCTGATTAGGCTCCAAC	
OCT4	Forward	GACAGGGGGAGGGGAGGAGCTAGG	2
	Reverse	CTTCCCTCCAACCAGTTGCCCCAAA	
PAX6	Forward	ATGTGTGAGTAAAATTCTGGGCA	3
	Reverse	GCTTACAACTTCTGGAGTCGCTA	
TUJ1	Forward	CCTGGAACCCGGAACCAT	2
	Reverse	AGGCCTGAAGAGATGTCCAAAG	
FOXG1	Forward	GCCACAATCTGTCCCTCAACA	4
	Reverse	CGGGTCCAGCATCCAGTAG	
IRX3	Forward	CCGTATGGCCAGTACCAGTT	4
	Reverse	ATAAGCGTTTCCCTCCTCGT	
GBX2	Forward	GGTGCAGGTGAAAATCTGGT	4
	Reverse	CCTGTCTTGGAATTGGCATT	
OTX1	Forward	GAGGTGGCGCTCAAGATCA	4
	Reverse	CGCGGCGGTTCTTGAA	
TBR1	Forward	ATGGGCAGATGGTGGTTTTA	4
	Reverse	GACGGCGATGAACTGAGTCT	
TBR2	Forward	CACCGCCACCAAACTGAGAT	4
	Reverse	CGAACACATTGTAGTGGGCAG	
NGN2	Forward	TGTTCGTCAAATCCGAGACCT	5
	Reverse	CGATCCGAGCAGCACTAACA	
EMX1	Forward	AGGTGAAGGTGTGGTTCCAG	4
	Reverse	AGTCATTGGAGGTGACATCG	
GSX2	Forward	CGCCACTTACCTGAACCTGT	4
	Reverse	CCCTCCTTCTTGTGCTTCAC	
DLX2	Forward	ACGCTCCCTATGGAACCAGTT	4
	Reverse	TCCGAATTTCAGGCTCAAGGT	
NKX2.1	Forward	AGCACACGACTCCGTTCTC	2
	Reverse	GCCCACTTTCTTGTAGCTTTCC	
VGLUT1	Forward	GAAACTCATGAACCCCCTCA	4
	Reverse	GGGAGATGAGCAGCAGGTAG	
VGLUT2	Forward	ATTCCATCAGCAGCCAGAGT	4
	Reverse	TTGCTCCATATCCCATGACA	
CASPASE3	Forward	CAAACTTTTTCAGAGGGGATCG	6
	Reverse	TGACACGCCATGTCATCATC	
GAD67	Forward	GCCAGACAAGCAGTATGATGT	4
	Reverse	CCAGTTCCAGGCATTTGTTGAT	
HB9	Forward	GCACCAGTTCAAGCTCAAC	7
	Reverse	GCTGCGTTTCCATTTCATCC	

ISL1	Forward	TGATGAAGCAACTCCAGCAG	8
	Reverse	GGACTGGCTACCATGCTGTT	
GFAP	Forward	CCCACTCTGCTTTGACTGAGC	2
	Reverse	CCTTCTTCGGCCTTAGAGGG	
GAPDH	Forward	CATGAGAAGTATGACAACAGCCT	3
	Reverse	AGTCCTTCCACGATACCAAAGT	

Supplementary Table S7 Primer sequences used in RT-PCR

Gene	Primer	Sequence	Reference
PDGFRα	Forward	CAGGTTGGTGTGGGTTCATT	9
	Reverse	AGGTGGGAGCATTTGTTAGG	
ISL1	Forward	TGATGAAGCAACTCCAGCAG	8
	Reverse	GGACTGGCTACCATGCTGTT	
AFP	Forward	ACCATGAAGTGGGTGGAATC	9
	Reverse	TGGTAGCCAGGTCAGCTAAA	
HNF1B	Forward	TCACAGATACCAGCAGCATCAGT	9
	Reverse	GGGCATCACCAGGCTTGTA	
PAX6	Forward	GTCCATCTTTGCTTGGGAAA	This study
	Reverse	CATTTGGCCCTTCGATTAGA	
MAP2	Forward	TGTGTCGTGTTCTCAAAGGG	10
	Reverse	TGCATATGCGCTGATTCTTC	
GAPDH	Forward	CACCAGGGCGCTTTTAACTCTG	11
	Reverse	ATGGTTCACACCCATGCGAAC	

References in Supplementary Information

1. Brennand, K. J. *et al.* Modelling schizophrenia using human induced pluripotent stem cells. *Nature* **473,** 221–225 (2011).
2. Zeng, L. *et al.* Functional Impacts of NRXN1 Knockdown on Neurodevelopment in Stem Cell Models. *PLoS One* **8,** (2013).
3. Sun, Y. *et al.* A deleterious Nav1.1 mutation selectively impairs telencephalic inhibitory neurons derived from Dravet Syndrome patients. *Elife* **5,** 1–27 (2016).
4. Espuny-Camacho, I. *et al.* Pyramidal Neurons Derived from Human Pluripotent Stem Cells Integrate Efficiently into Mouse Brain Circuits In Vivo. *Neuron* **77,** 440–456 (2013).
5. Pawlowski, M. *et al.* Inducible and Deterministic Forward Programming of Human Pluripotent Stem Cells into Neurons, Skeletal Myocytes, and Oligodendrocytes. *Stem Cell Reports* **8,** 803–812 (2017).
6. Li, F. *et al.* Apoptotic caspases regulate induction of iPSCs from human fibroblasts. *Cell Stem Cell* **7,** 508–520 (2010).
7. Alves, C. J. *et al.* Gene expression profiling for human iPS-derived motor neurons from sporadic ALS patients reveals a strong association between mitochondrial functions and neurodegeneration. *Front. Cell. Neurosci.* **9,** (2015).
8. Li, W. *et al.* Characterization and transplantation of enteric neural crest cells from human induced pluripotent stem cells. *Mol. Psychiatry* **23,** 499–508 (2018).
9. Chng, Z., Teo, A., Pedersen, R. A. & Vallier, L. SIP1 Mediates Cell-Fate Decisions between Neuroectoderm and Mesendoderm in Human Pluripotent Stem Cells. *Cell Stem Cell* **6,** 59–70 (2010).
10. Malik, N. *et al.* Comparison of the gene expression profiles of human fetal cortical astrocytes with pluripotent stem cell derived neural stem cells identifies human astrocyte markers and signaling pathways and transcription factors active in human astrocytes. *PLoS One* **9,** (2014).
11. Yokoo, N. *et al.* The effects of cardioactive drugs on cardiomyocytes derived from human induced pluripotent stem cells. *Biochem. Biophys. Res. Commun.* **387,** 482–488

Figure S1

A

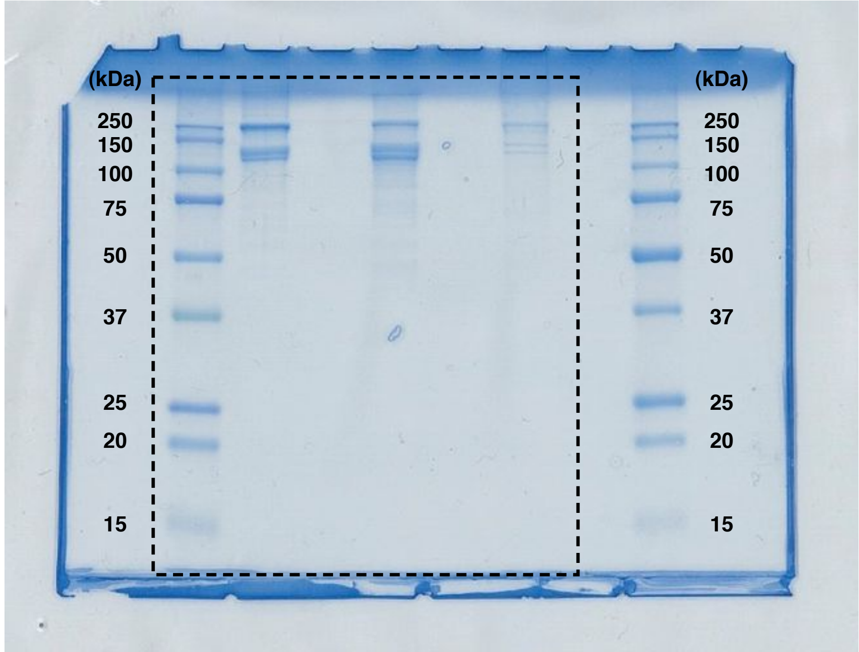

B a

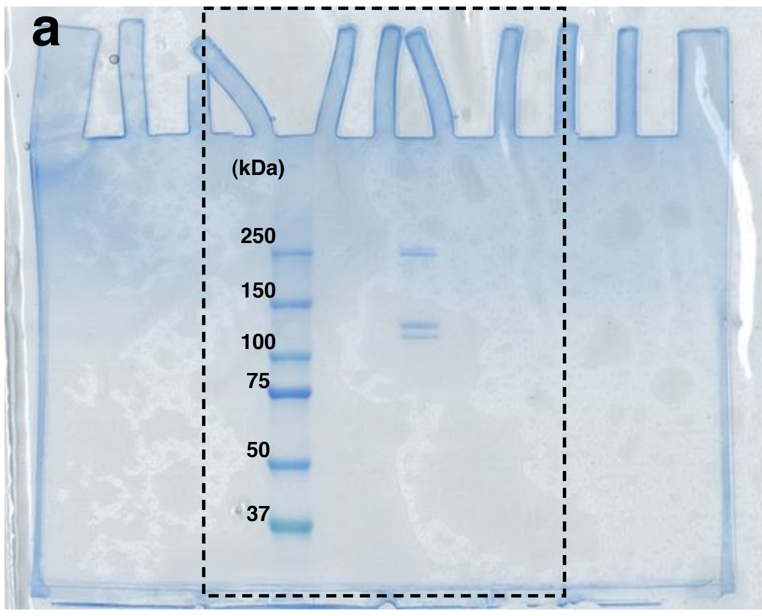

b

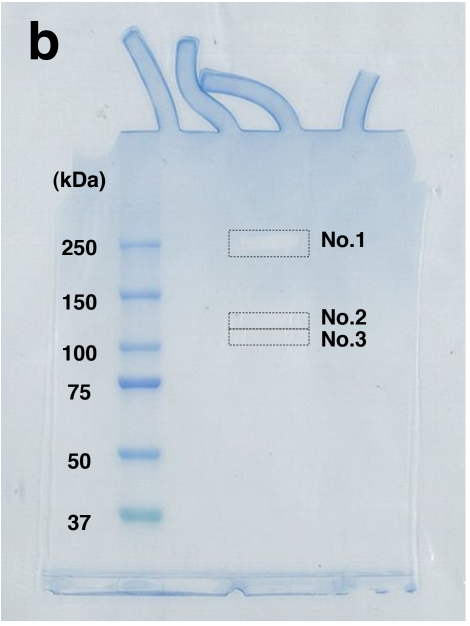

C

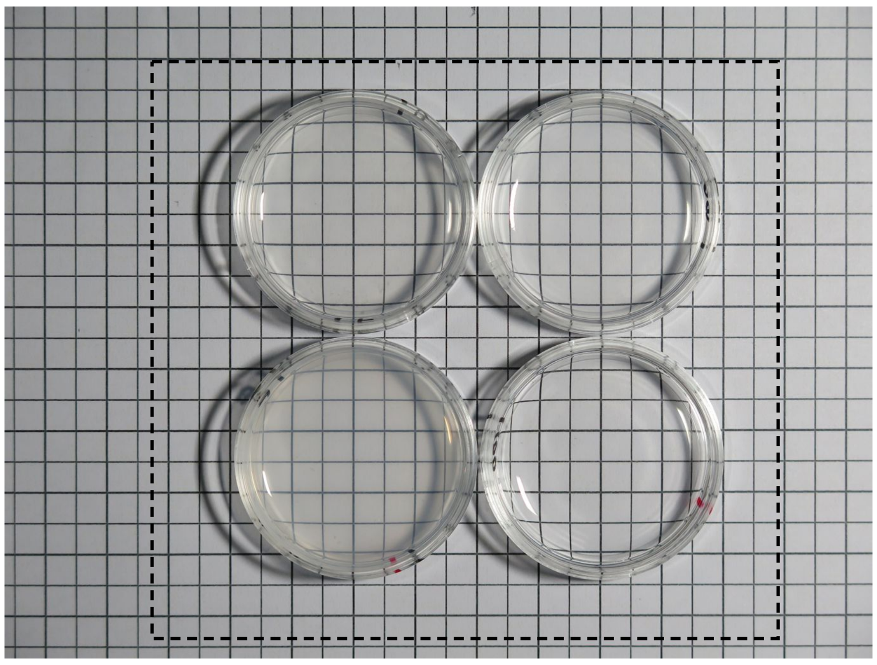

Figure S2

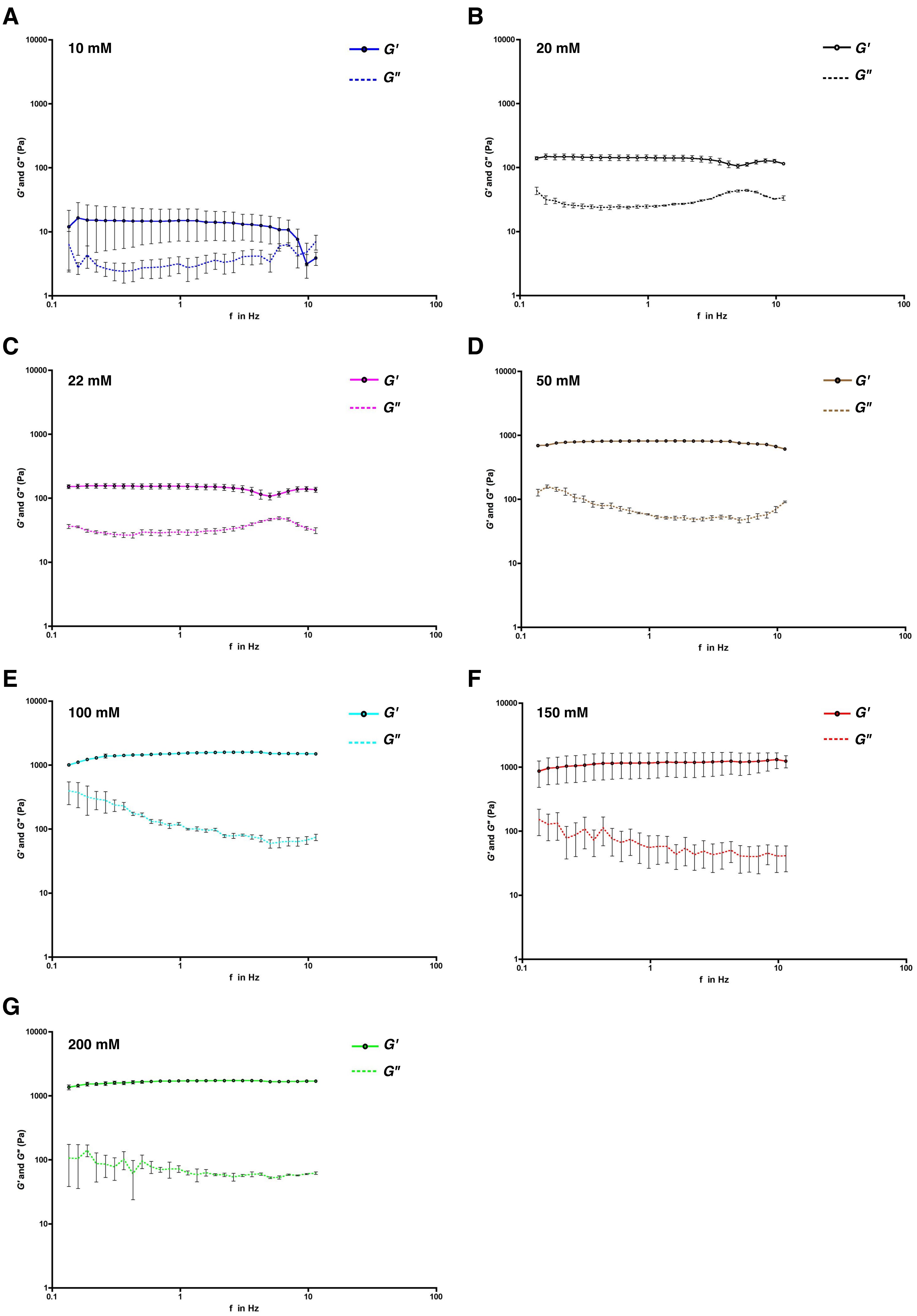

A
10 mM
G'
G"
B
20 mM
G'
G"
C
22 mM
G'
G"
D
50 mM
G'
G"
E
100 mM
G'
G"
F
150 mM
G'
G"
G
200 mM
G'
G"
G' and G" (Pa)
f in Hz

Figure S3

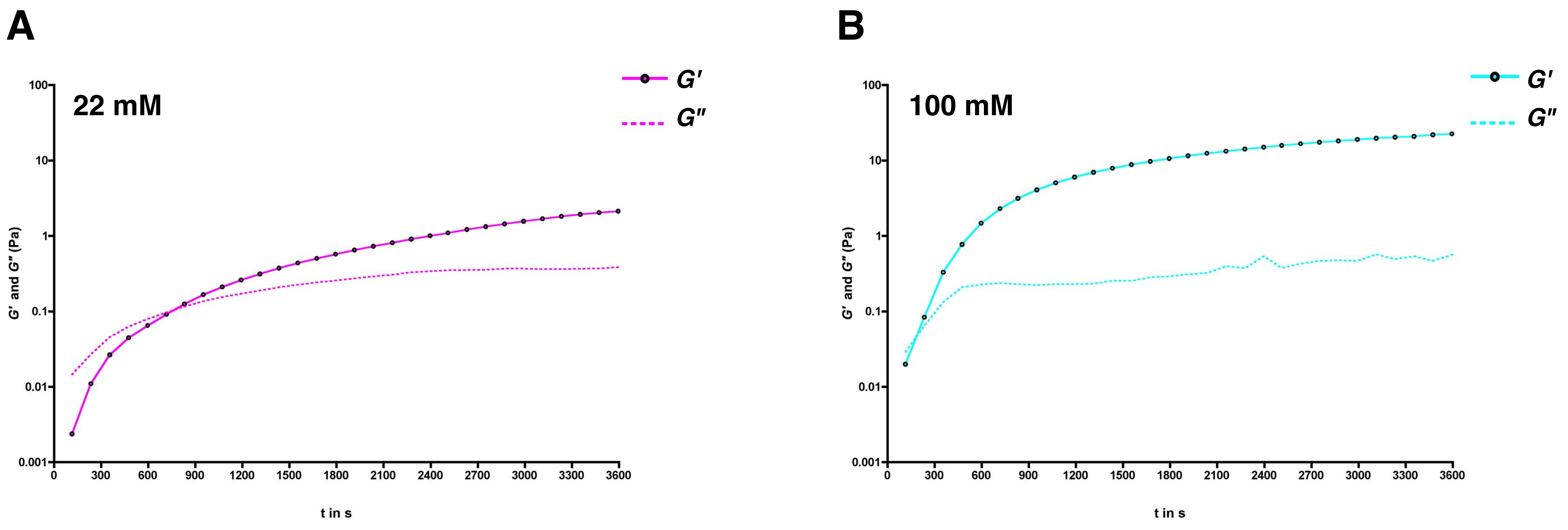
A
22 mM
G′
G″
G′ and G″ (Pa)
100
10
1
0.1
0.01
0.001
0
300
600
900
1200
1500
1800
2100
2400
2700
3000
3300
3600
t in s
B
100 mM
G′
G″
G′ and G″ (Pa)
t in s

Figure S4

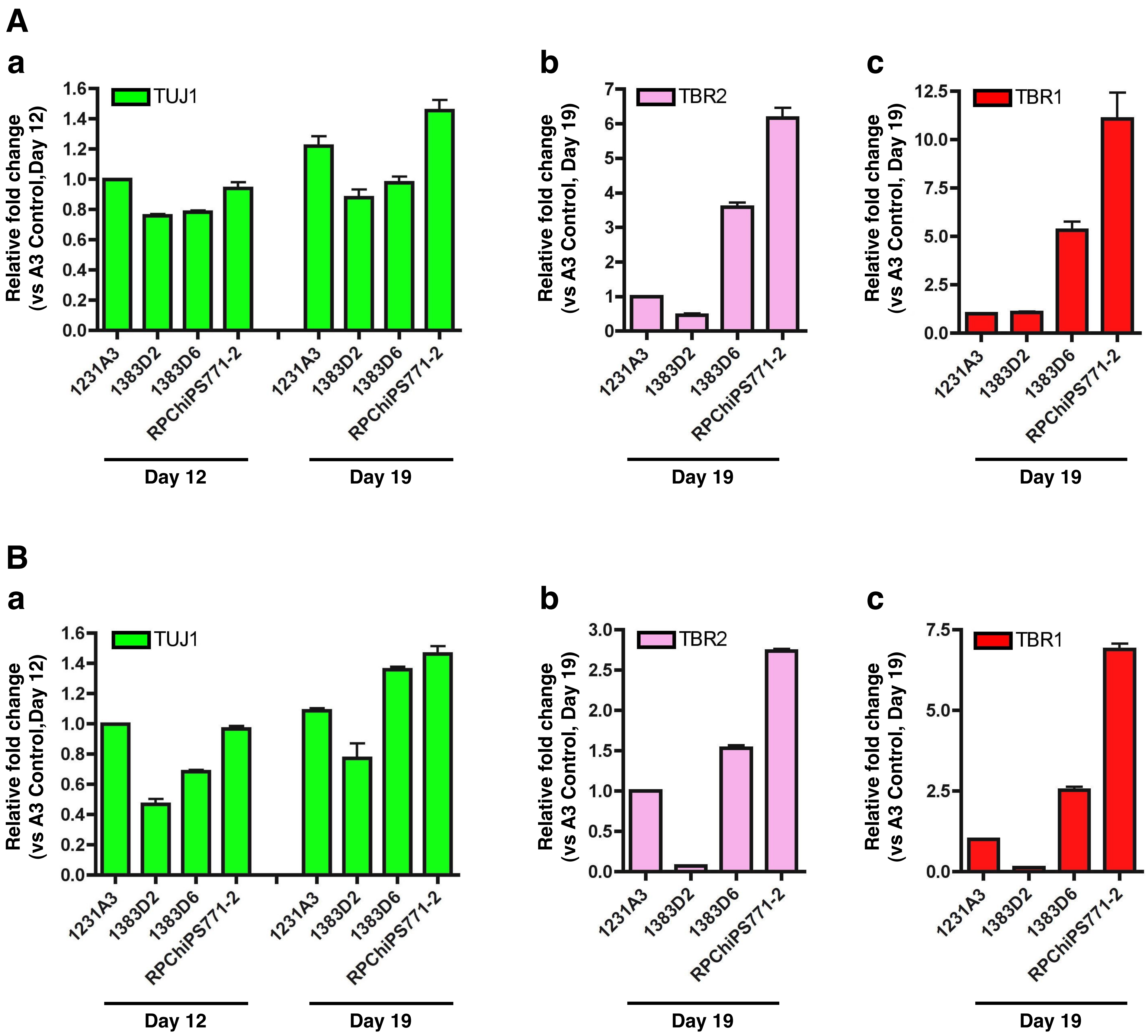
A
a
TUJ1
Relative fold change
(vs A3 Control,Day 12)
1231A3
1383D2
1383D6
RPChiPS771-2
Day 12
Day 19
b
TBR2
Relative fold change
(vs A3 Control, Day 19)
c
TBR1
B

Figure S5

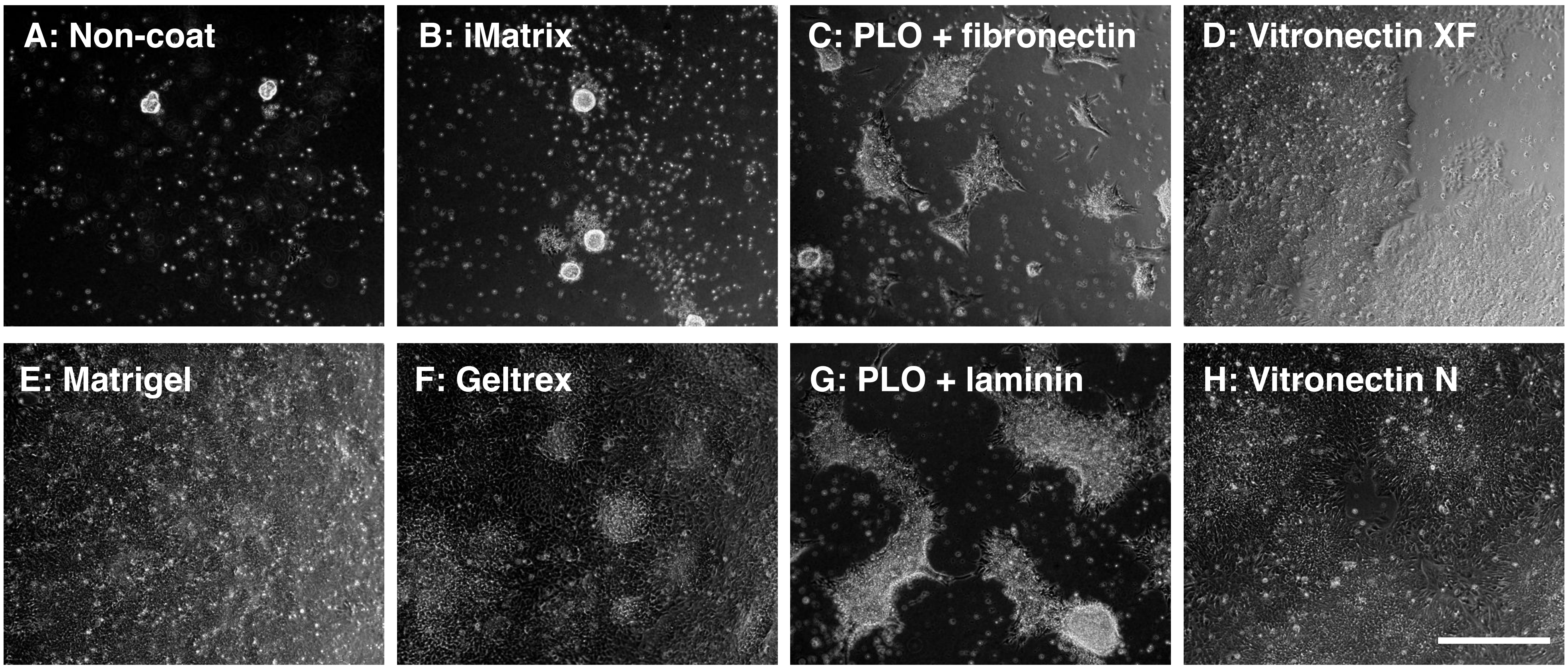
A: Non-coat
B: iMatrix
C: PLO + fibronectin
D: Vitronectin XF
E: Matrigel
F: Geltrex
G: PLO + laminin
H: Vitronectin N

Figure S6

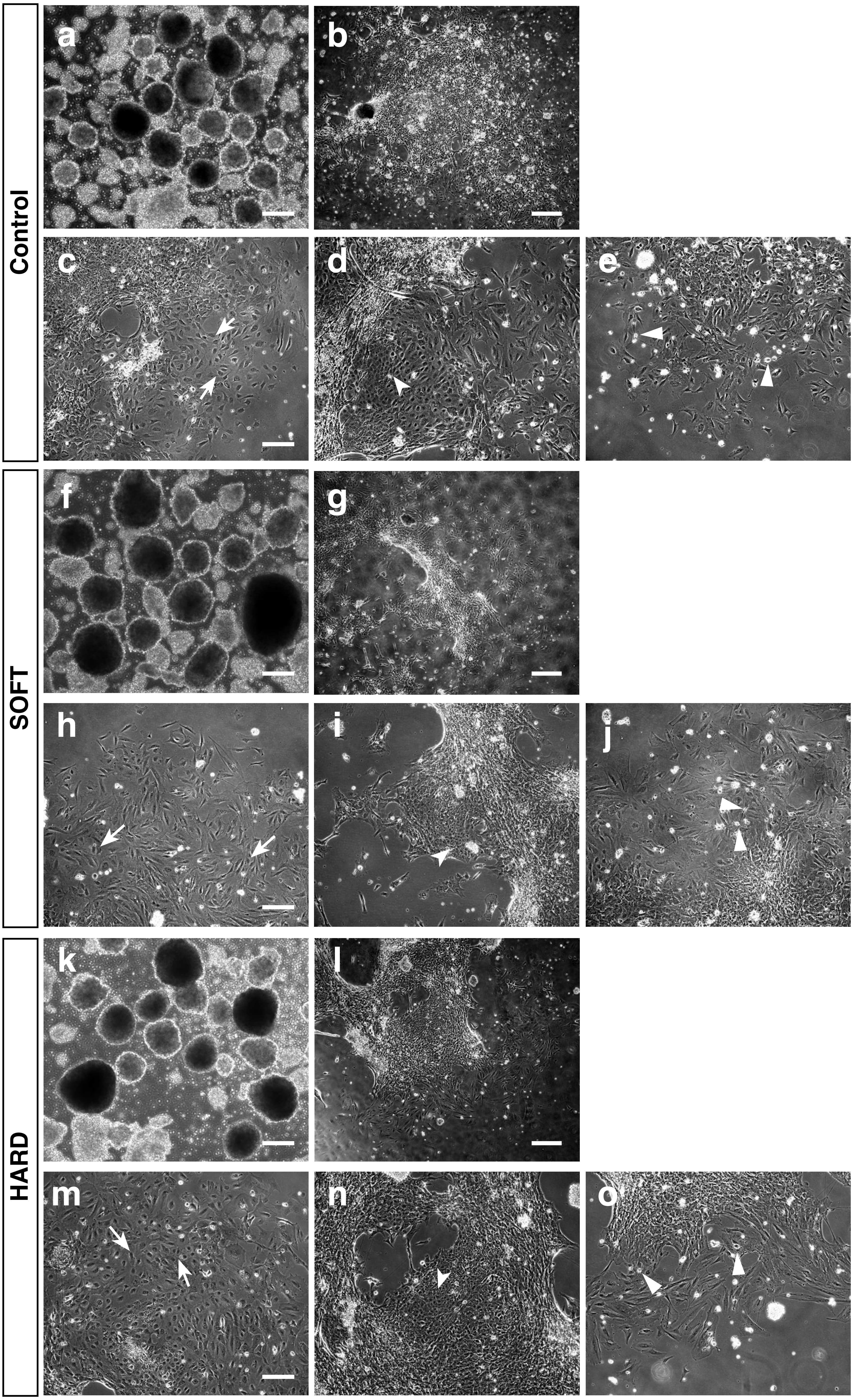

Figure S7

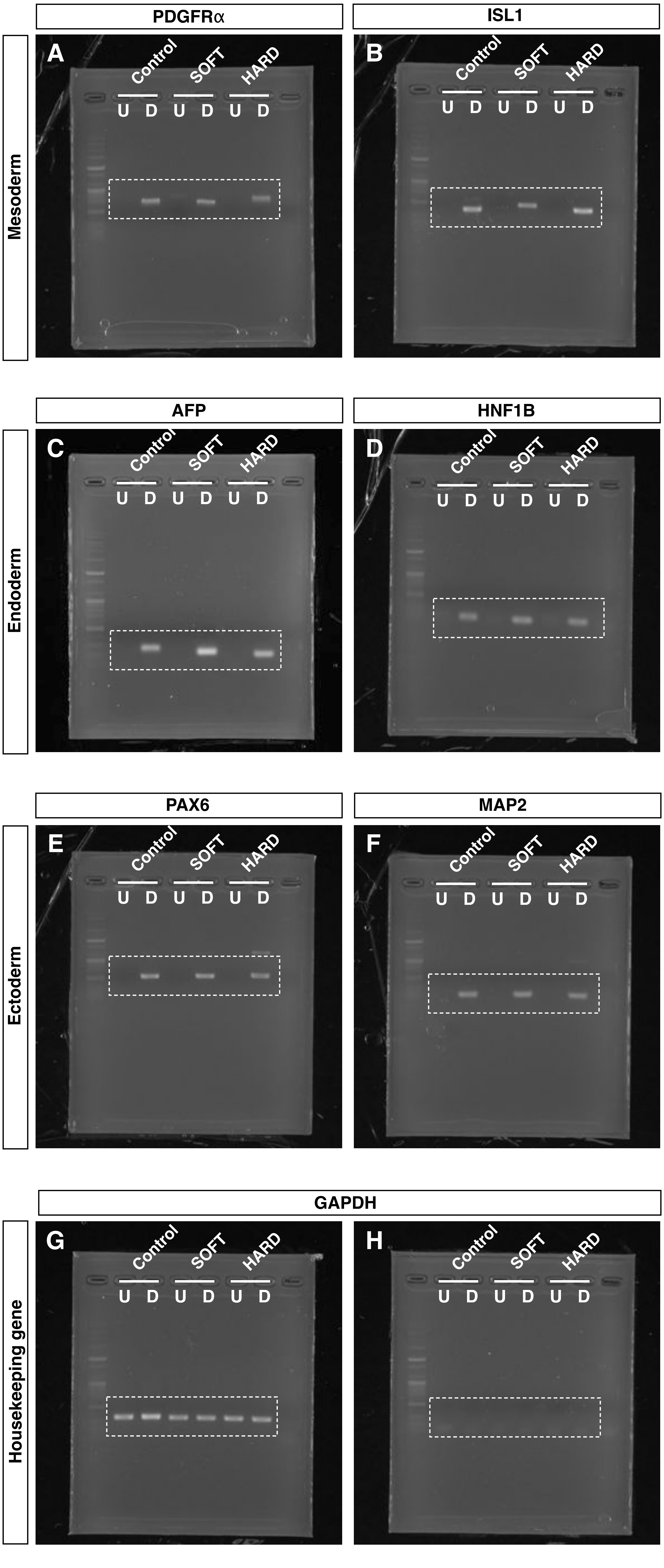
PDGFRα
ISL1
Mesoderm
A
Control
SOFT
HARD
U D U D U D
B
AFP
HNF1B
Endoderm
C
D
PAX6
MAP2
Ectoderm
E
F
GAPDH
Housekeeping gene
G
H

Figure S8

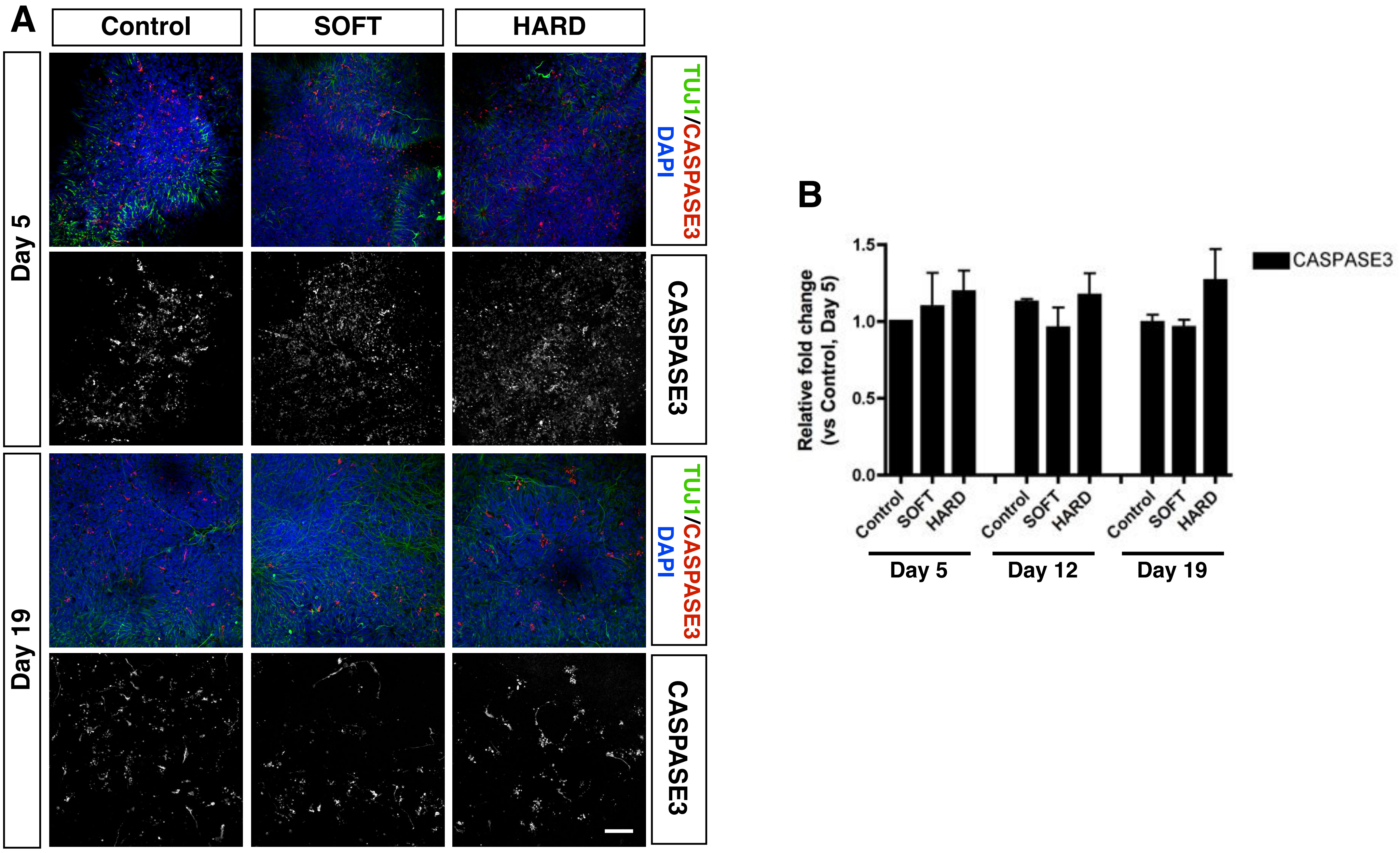
A
Control
SOFT
HARD
Day 5
Day 19
TUJ1/CASPASE3
DAPI
CASPASE3
B
Relative fold change
(vs Control, Day 5)
1.5
1.0
0.5
0.0
CASPASE3
Control
SOFT
HARD
Day 5
Day 12
Day 19

Figure S9

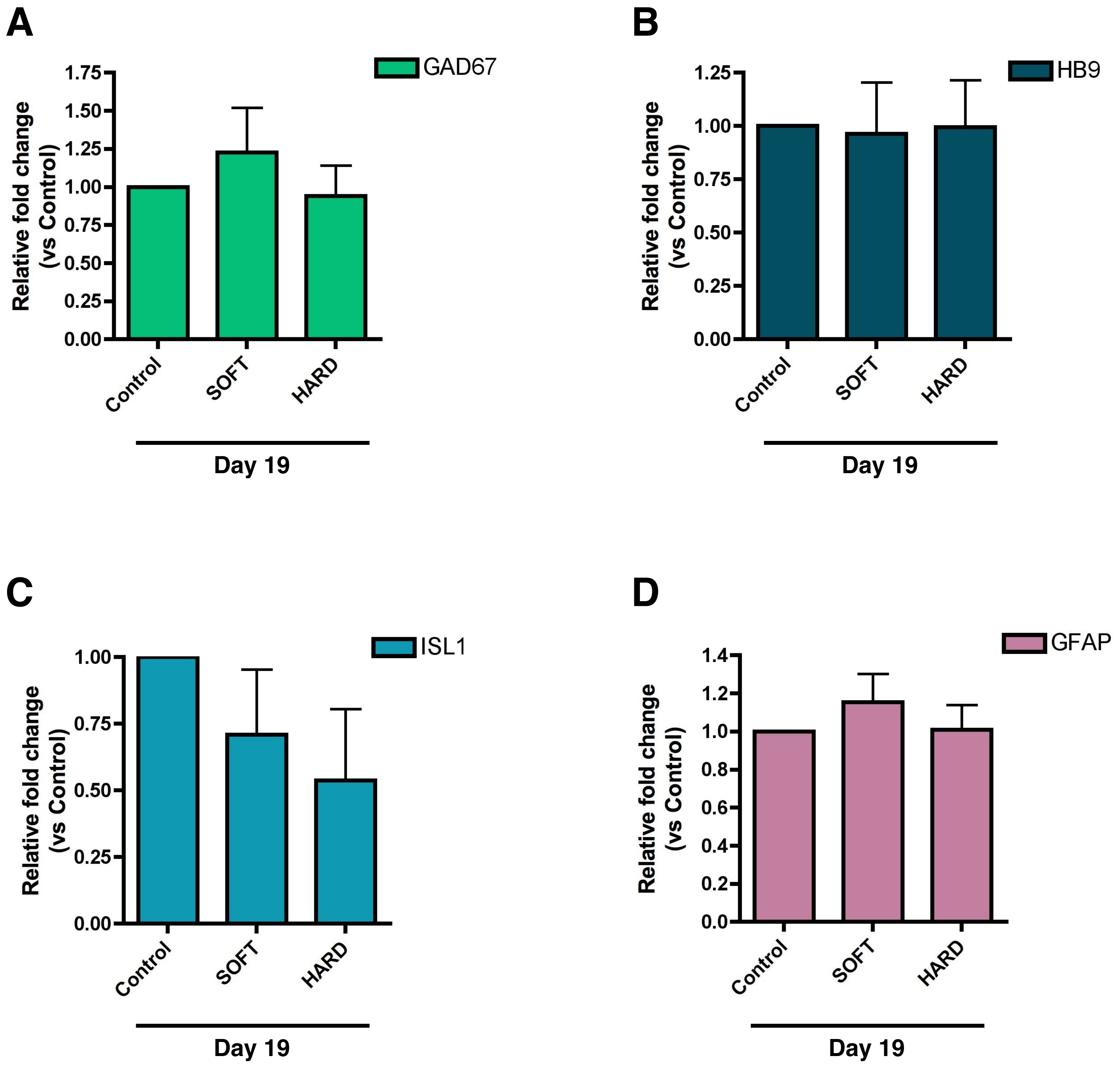
A
GAD67
Relative fold change (vs Control)
1.75
1.50
1.25
1.00
0.75
0.50
0.25
0.00
Control
SOFT
HARD
Day 19
B
HB9
Relative fold change (vs Control)
1.25
1.00
0.75
0.50
0.25
0.00
Control
SOFT
HARD
Day 19
C
ISL1
Relative fold change (vs Control)
1.00
0.75
0.50
0.25
0.00
Control
SOFT
HARD
Day 19
D
GFAP
Relative fold change (vs Control)
1.4
1.2
1.0
0.8
0.6
0.4
0.2
0.0
Control
SOFT
HARD
Day 19

Figure S10

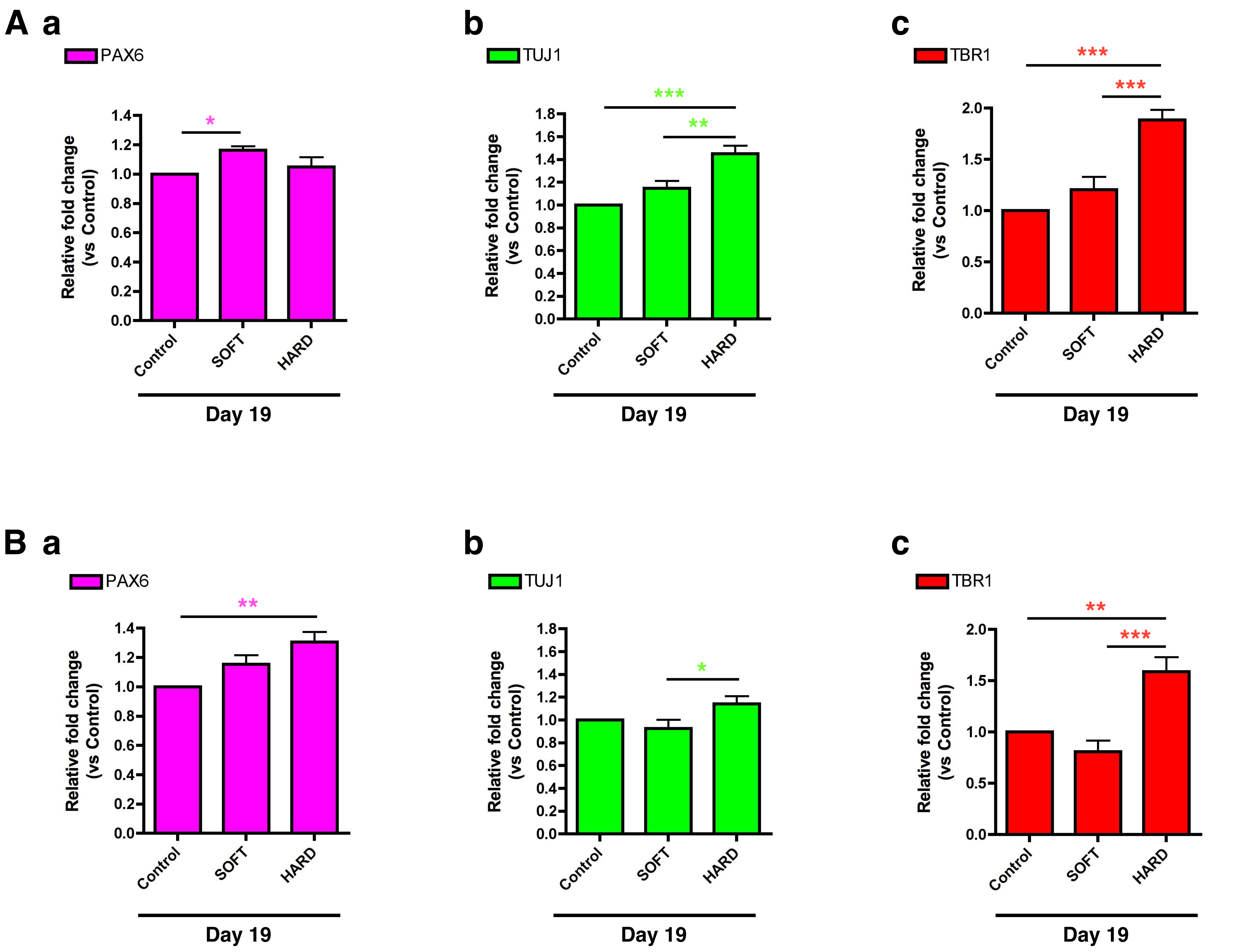
A a
PAX6
*
Relative fold change (vs Control)
1.4
1.2
1.0
0.8
0.6
0.4
0.2
0.0
Control
SOFT
HARD
Day 19
b
TUJ1
***
**
1.8
1.6
c
TBR1
***
2.0
1.5
0.5
B a
**
b
*
c

Figure S11

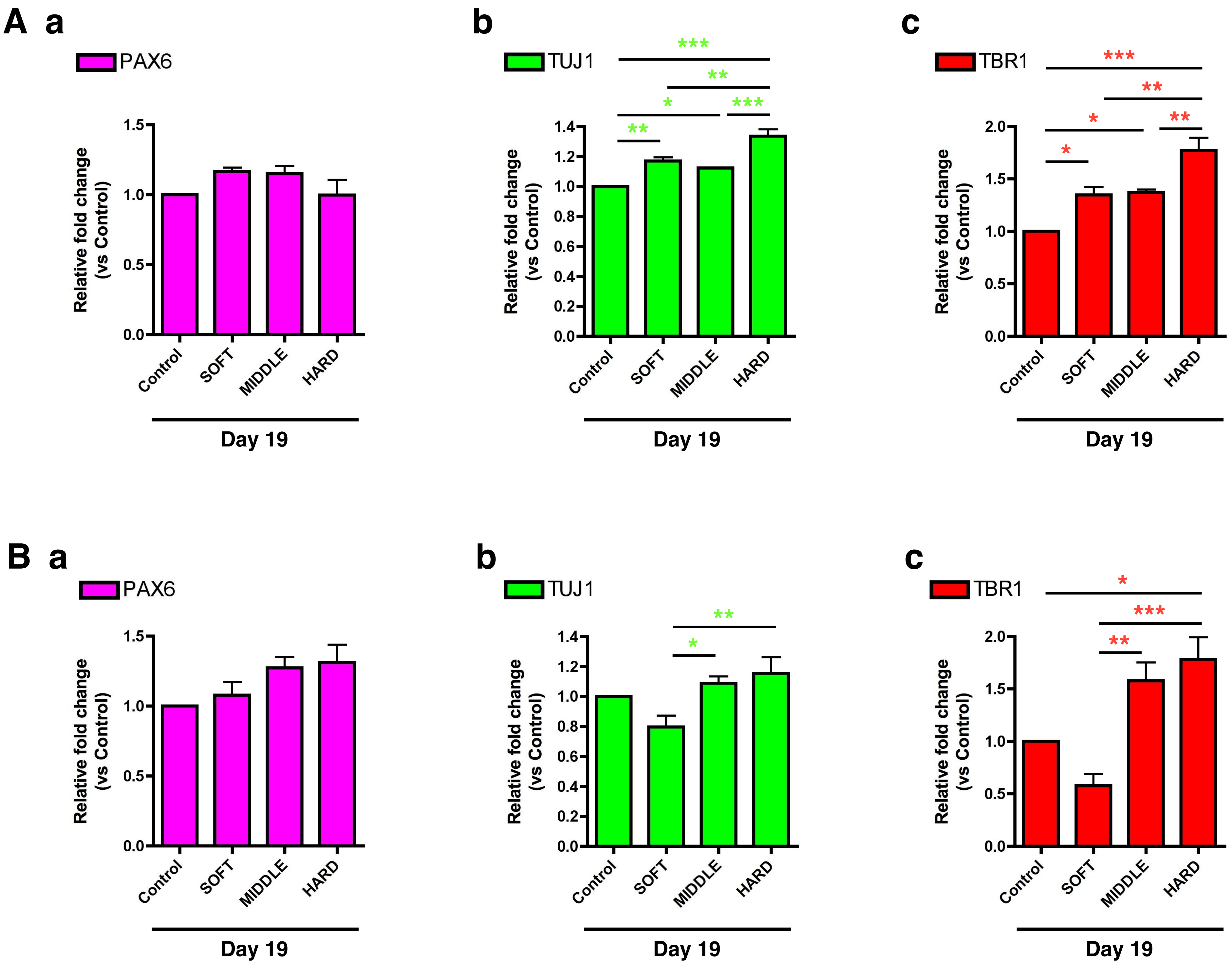
A a
PAX6
Relative fold change (vs Control)
1.5
1.0
0.5
0.0
Control
SOFT
MIDDLE
HARD
Day 19
b
TUJ1
***
**
*
***
**
1.4
1.2
1.0
0.8
0.6
0.4
0.2
0.0
c
TBR1
***
**
*
**
*
2.0
1.5
1.0
0.5
0.0
B a
PAX6
b
TUJ1
**
*
c
TBR1
*
***
**

Figure S12

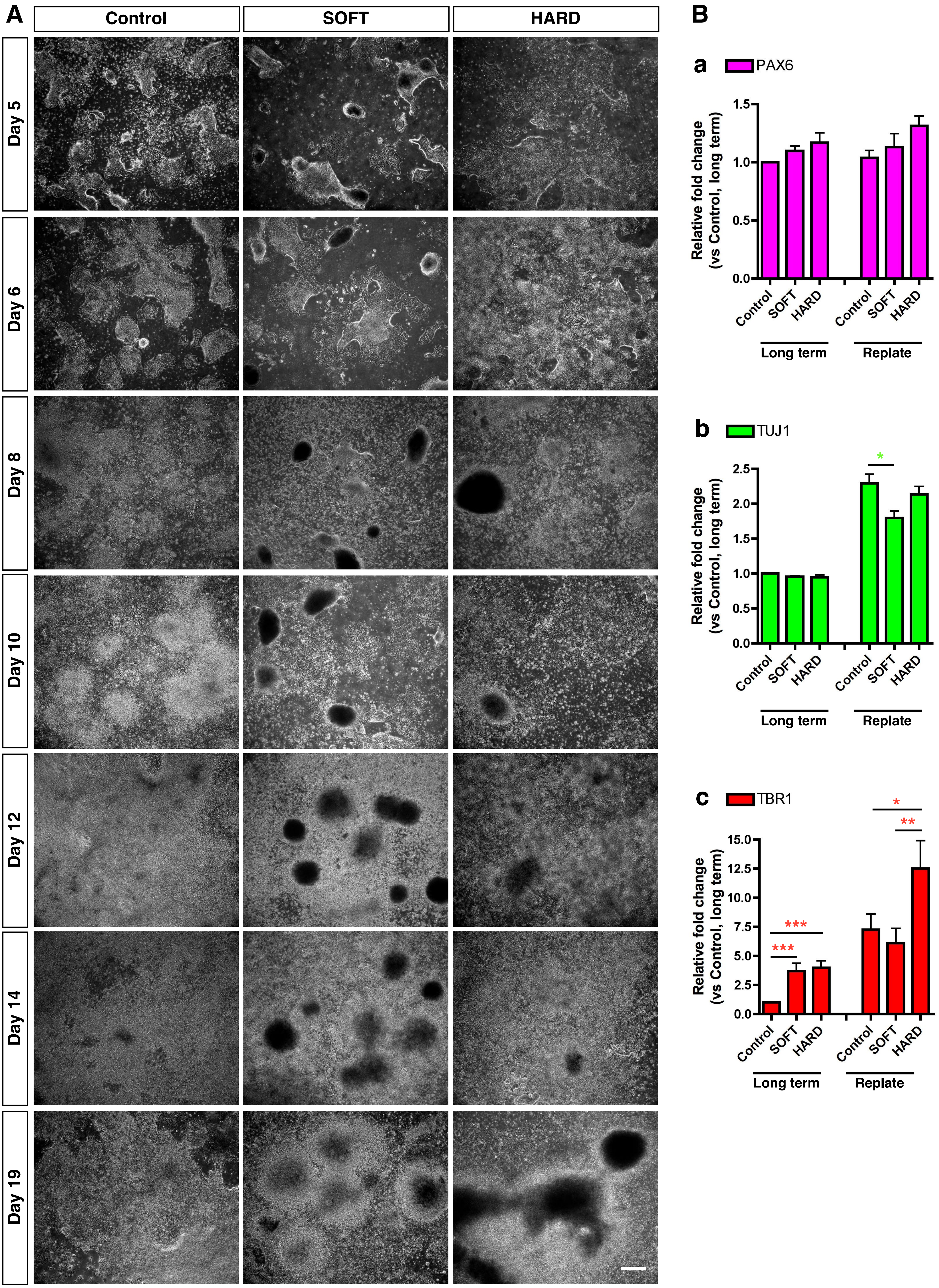

A
Control
SOFT
HARD
Day 5
Day 6
Day 8
Day 10
Day 12
Day 14
Day 19
B
a
PAX6
Relative fold change (vs Control, long term)
1.5
1.0
0.5
0.0
Control
SOFT
HARD
Long term
Replate
b
TUJ1
*
2.5
2.0
1.5
1.0
0.5
0.0
c
TBR1
*
**
***
***
15.0
12.5
10.0
7.5
5.0
2.5
0.0
